# The illusion of progress: why most introgressions from wild Triticeae fail to improve wheat agronomically

**DOI:** 10.3389/fpls.2026.1886864

**Published:** 2026-07-03

**Authors:** Pavel Yu. Kroupin, Anna I. Yurkina, Maida Jazmin González Franco, Viktoria M. Sokolova, Gennady I. Karlov, Mikhail G. Divashuk

**Affiliations:** Department of Applied Genetic Technologies, All-Russia Research Institute of Agricultural Biotechnology, Moscow, Russia

**Keywords:** breeding constraints, field validation, genetic resources, introgression, linkage drag, polygenic traits, *Triticum aestivum*, wild Triticeae

## Abstract

The progressive narrowing of the genetic base of bread wheat (*Triticum aestivum* L.) threatens yield gains and stress resilience under climate change. Wild Triticeae species are widely viewed as a vital source of adaptive diversity, yet despite decades of research and the discovery of numerous genes and introgression lines, only a tiny fraction has delivered validated agronomic benefits. Because introgression from the secondary gene pool (*Aegilops* spp.) follows distinct genetic rules, this review deliberately focuses on more distant genera within the tertiary gene pool. This review critically dissects that disconnect, revealing a systematic “illusion of progress.” We demonstrate how methodological biases—particularly reliance on surrogate traits under controlled conditions and insufficient multi-environment validation—interact with genetic constraints such as linkage drag, polygenic architecture, and segment instability to inflate early-stage promise while suppressing field-level reproducibility. By contrasting successful and unsuccessful introgressions, we identify the conditions essential for translational impact: major-effect loci, minimal linkage drag, stable expression across environments, and rigorous field testing. To bridge the lab-to-field gap, we advocate for agronomically relevant phenotyping, precision introgression including genome editing, integration with genomic selection, and a fundamental shift from reporting gene-level discoveries to demonstrating measurable yield performance. This work provides a conceptual framework for evaluating introgression outcomes and offers actionable strategies to improve the efficiency of exotic gene transfer in wheat breeding, thereby addressing a central challenge in plant breeding and food security.

## 1 Introduction

The progressive narrowing of the genetic base of bread wheat (*Triticum aestivum* L.), driven by intensive breeding and the widespread adoption of high-yielding cultivars, has become a major constraint for further genetic improvement. Though patterns vary by crop and region, this genetic erosion limits the availability of allelic variation required to enhance resistance to emerging pathogens and tolerance to abiotic stresses, particularly under rapidly changing climatic conditions (Reif et al., 2005; Van De Wouw et al., 2010; Khoury et al., 2022). In this context, wild relatives of wheat, especially species within the tribe Triticeae, are widely recognized as an important reservoir of adaptive genetic diversity (Kumar et al., 2022; Ortiz et al., 2024; Tian et al., 2026).

Introgression, defined as the transfer of genetic material from wild species into the genome of cultivated wheat, has long been considered a key strategy for broadening the genetic base of breeding material (Friebe et al., 1996; Warschefsky et al., 2014; Boehm and Cai, 2024; Blower et al., 2025). Over the past decades, numerous studies have reported the identification of genes, quantitative trait loci (QTLs), and introgression lines associated with resistance to diseases, salinity, drought, and other stress factors (Choudhary et al., 2017; Kishii, 2019; Kuzmanović et al., 2021; Wang et al., 2025c). Advances in cytogenetic techniques, molecular markers, and more recently genomic and transcriptomic tools have further accelerated the detection and characterization of potentially valuable alleles derived from genera such as *Thinopyrum*, *Dasypyrum*, *Elymus*, *Pseudoroegneria*, *Roegneria*, and *Leymus* (Divashuk et al., 2022; Keilwagen et al., 2023; Kroupin et al., 2023).

However, despite the extensive body of research and the large number of reported introgressions, only a small proportion has resulted in commercially relevant cultivars or demonstrated consistent agronomic benefits under field conditions (Hajjar and Hodgkin, 2007; Dempewolf et al., 2017; Ortiz et al., 2024; Khalid et al., 2025). This discrepancy between the volume of published data and its practical impact highlights systemic limitations that remain insufficiently addressed in the current literature.

Consequently, the central question is not whether wild relatives harbor useful genetic variation, but under which conditions introgressed material can contribute to reproducible and agronomically meaningful improvements in wheat performance. In particular, it remains unclear why the majority of reported introgressions fail to progress beyond experimental systems and do not result in widely adopted cultivars.

The aim of this review is to provide a critical analysis of recent advances in the use of wild Triticeae species for the introgressive improvement of bread wheat, with a focus on identifying the key factors limiting their practical application in breeding. Because *Aegilops* species are phylogenetically closer to bread wheat and generally exhibit higher homoeologous recombination rates with significantly lower linkage drag, their introgression follows different genetic rules and is comparatively less constrained. Therefore, this review deliberately focuses on more distant Triticeae genera (e.g., *Thinopyrum, Dasypyrum, Elymus, Leymus, Roegneria, Pseudoroegneria*) to highlight constraints specific to tertiary gene pool transfers. For a comparative baseline, we briefly reference well−documented *Aegilops*−derived successes (e.g., *Lr34, Lr42*) in the discussion, but a full analysis of *Aegilops* is beyond our scope. This exclusion ensures a clearer focus on the limitations associated with more distant relatives, which are the primary source of translational failure discussed here.

Specifically, we examine methodological constraints in trait evaluation, genetic limitations associated with introgression, and the discrepancy between reported gene effects and field performance. By integrating evidence from both successful and unsuccessful cases, this review seeks to define the conditions under which introgression is most likely to be effective and to outline strategies for improving its translational potential in modern wheat breeding.

Contribution statement: unlike previous reviews that primarily summarize available genetic resources and reported introgressions, this study focuses on identifying the key factors limiting their effective use in wheat breeding. By systematically analyzing both successful and unsuccessful cases, we highlight the methodological and genetic constraints that prevent the translation of introgressed variation into stable agronomic gains. This work provides a conceptual framework for evaluating introgression outcomes and outlines criteria for improving their practical relevance in breeding programs.

## 2 Methods

This review is designed as a critical narrative synthesis with systematic elements. A structured literature search was conducted and explicit inclusion/exclusion criteria were applied, but the primary goal was conceptual synthesis rather than a formal systematic review with meta−analysis.

### 2.1 Search strategy

We searched PubMed, Web of Science Core Collection, Google Scholar, and the AGRIS database for peer−reviewed articles published between January 1968 and March 2026. The following search strings were used (adapted to the syntax of each database): (*Triticum aestivum* or wheat or bread wheat) and (introgression or alien introgression or translocation or alien chromatin or chromosome engineering) and (*Thinopyrum* or *Agropyron* or *Lophopyrum* or *Dasypyrum* or *Haynaldia* or *Elymus* or *Leymus* or *Psathyrostachys* or *Roegneria* or *Pseudoroegneria* or *Tritipyrum* or *Trititrigia* or «wild relative»). Additional targeted searches were performed for specific genes or loci: *Pm21*, *Fhb1*, *Fhb7*, *Lr19*, *Lr55*, *Sr24*, *Sr25*, *Sr26*, *Sr43*, *Sr68*, *Wsm1*, *Bdv2*, *Yr4EL*, *Yr69*, *CreV*, *Pm62*, *Pm67*, and 6Ag^i^2. The bibliographies of retrieved articles and key reviews were also screened to identify any relevant studies that might have been missed by the electronic search. The combined searches retrieved approximately 1,300 records. After removing duplicates and screening titles and abstracts, about 250 full−text articles were assessed for eligibility. Of these, approximately 75 studies, together with additional references identified through bibliography screening, provided the data on the 70 unique alien introgressions that form the basis of the quantitative synthesis (**Supplementary Table 4**).

### 2.2 Inclusion and exclusion criteria

Studies were included if they met all of the following criteria: (i) successful transfer of alien chromatin from a wild Triticeae species (excluding *Aegilops*, except as noted in **Supplementary Table 1**) into bread wheat; (ii) at least minimal field validation (one location, one growing season) or documented commercial or regional deployment; (iii) reporting of agronomic performance data (yield, yield components, or disease resistance under field conditions). Purely molecular or *in vitro* studies without wheat introgression were excluded, as were studies that did not provide original data (e.g., opinion pieces, conference abstracts without full data). For widely cited loci that lack full field validation (e.g., *Yr69, CreV*), we included them in Tier 3 as illustrative examples of weak evidence, following the same logic applied throughout the review.

### 2.3 Screening and data extraction

Titles and abstracts were screened independently by two reviewers. Full−text versions of potentially eligible records were retrieved. Disagreements about inclusion were resolved by consensus, with a third reviewer consulted if no agreement could be reached. From each included study, the following information was extracted: donor species, introgressed segment or gene, target trait, field validation level (High, Medium, Low), yield effect, linkage drag, deployment status, and key limitations. For the purpose of this inventory, we considered not only stable introgression lines carrying a defined alien gene or segment, but also chromosome addition, substitution, and translocation stocks, as well as partial amphiploids, when the original authors explicitly presented them as sources of traits for potential transfer into bread wheat. The tier classification reflects the level of agronomic validation of the trait in that germplasm, irrespective of its cytogenetic type. Purely molecular or in silico candidates without any field validation were excluded.

### 2.4 Synthesis and classification

Because the aim of this review was a critical narrative synthesis rather than a formal meta−analysis, we did not pool effect sizes statistically. Instead, we used a qualitative tier system to assess the translational relevance of each introgression (see Main **Table 1**; **Supplementary Table 2**). The classification criteria were defined *a priori*:

- Tier 1: High field validation (≥3 environments, ≥2 years) + commercial deployment + evidence from at least two independent studies.
- Tier 2: Medium field validation (≤2 environments or ≤2 years) + pre−breeding or regional deployment.
- Tier 3: Low field validation (controlled or single−environment studies only) or a single published report.

**Table 1:** Representative introgressions from wild Triticeae into bread wheat: agronomic impact, validation status, and key limitations.

Gene/locus	Trait	Donor species	Genetic unit	Tier	Field validation	Evidence strength	Deployment status	Yield effect	Linkage drag	Main limitation(s)	Key reference(s)
*Lr24/Sr24*	Leaf/stem rust	*Th. ponticum*	Locus (mapped)	1	High	Strong	Commercial (historical)	Neutral (background−dependent)	High (reduced via recombination)	Initial linkage drag (red grain); *Sr24* ineffective against TTKST	(Smith et al., 1968; Plotnikova et al., 2023; Li et al., 2024b)
*Lr19*	Leaf rust	*Th. ponticum*	Locus (mapped)	1	High	Strong	Commercial (historical/regional)	Neutral (in recombinants)	High (reduced)	Yellow pigment linkage (*PSY-E1*); yield penalty in some backgrounds	(Knott, 1968; Xu et al., 2023)
*Lr55*	Leaf rust	*E. trachycaulus*	Locus (mapped)	3	Low	Weak	Pre-breeding	Not evaluated	High	Not cloned; linked markers are distant; original whole-arm alien segment; no field yield validation	(Friebe et al., 2005; Pietrusińska and Tyrka, 2021; Wang et al., 2025d)
*Sr25*	Stem rust	*Th. ponticum*	Locus (mapped)	2	Medium	Moderate	Pre−breeding	Neutral (background−dependent)	High	Yellow pigment linkage; yield penalty reported in some backgrounds	(Liu et al., 2010; Plotnikova et al., 2023; GrainGenes, n.d.)
*Sr26*	Stem rust	*Th. ponticum*	Locus (mapped)	1	Medium	Moderate	Commercial (Australia)	Neutral (modern recombinants)	High (reduced)	Initial 9% yield penalty eliminated via recombination	(Dundas et al., 2007, 2015)
*Sr43*	Stem rust	*Thinopyrum* spp.	Gene (cloned)	2	Medium	Moderate	Pre−breeding	Not evaluated	Low (engineered)	Not yet deployed; encodes unusual protein kinase; original large segment; yellow pigment linkage reduced but not fully eliminated	(Niu et al., 2014; Yu et al., 2023)
*Sr52*	Stem rust	*D. villosum*	Locus (mapped)	3	Low	Weak	Pre-breeding	Not evaluated	Low (engineered 8% distal segment)	Not cloned; single-study physical mapping; no field/yield validation	(Li et al., 2019)
*Pm21*	Powdery mildew	*D. villosum*	Gene (cloned)	1	High	Strong	Commercial (China>4M ha)	Positive (under disease pressure)	Low	Race specificity risk; widely deployed	(Xing et al., 2018; Ye et al., 2019)
*Pm55*	Powdery mildew	*D. villosum*	Gene (cloned)	2	Medium	Moderate	Pre−breeding	Neutral	Low	Developmental−stage specific; limited field validation	(Lu et al., 2024)
*Fhb6*	FHB	*E. tsukushiensis*	Locus	3	Low	Weak	Pre−breeding	Not evaluated	Weak	Gene not cloned; exact segment size not reported; limited validation; no yield data	(Cainong et al., 2015)
*Fhb7*	FHB	*Th. ponticum*	Gene (GST, HGT)	2	Medium	Moderate	Pre−commercial	Neutral (in recombinants)	High (broken in specific alleles)	Linked to *PSY-E2* (broken in specific alleles (Li et al., 2023)); HGT from fungus; background−dependent effects; validated in NILs without yield penalty	(Guo et al., 2015, 2023b; Wang et al., 2020; Li et al., 2023)
*Fhb-7EL*	FHB	*Th. elongatum*	Locus (QTL)	3	Medium	Moderate	Pre−breeding	Neutral	Low-Moderate	Major locus with strong effect; deployed in specific backgrounds; relationship to *Fhb7* unclear (see footnote)	(Guo et al., 2023a)
*Wsm1*	WSMV	*Th. intermedium*	Locus (mapped)	1	High	Strong	Commercial (USA)	Negative (original T4DL·4JsS, virus free) → Positive (rec213, ≤12.6% vs. near-isogenic sibs without translocation, virus-free conditions)	High (reduced in rec213)	Temperature−sensitive (>20 °C); resistance−breaking strains; rec213 shows yield benefit up to 12.6%	(Wells et al., 1982; Sharp et al., 2002; Guttieri et al., 2023)
*Bdv2*	BYDV	*Th. intermedium*	Locus (mapped)	2	Medium	Moderate	Commercial (Europe, Australia, China and Mexico)	Neutral to positive (under pressure)	Moderate	Large segment; limited multi−environment yield data	(Larkin et al., 2002; Gao et al., 2009; Silva et al., 2022; Qonaah et al., 2026)
*Yr69*	Yellow rust	*Th. ponticum*	Locus	3	Low	Weak	None	Not evaluated	Unknown	QTL not cloned; no multi−environment field validation	(Hou et al., 2016)
*YrTp1/YrTp2*	Yellow rust	*Th. ponticum*	Locus	3	Low	Weak	None	Not evaluated	Unknown	Single−study loci; no independent validation	(Yin et al., 2006)
*CreV*	Cyst nematode	*D. villosum*	Locus	3	Low	Weak	None	Not evaluated	Low	Greenhouse−only validation; no field yield data	(Zhang et al., 2016)
*6Ag^i^2* (6D)	Disease complex	*Th. intermedium*	Segment	2	Medium	Moderate	Regional (Russia)	Neutral	Medium	Stable resistance; limited multi−environment yield validation; no penalty reported	(Salina et al., 2015; Ivanova et al., 2021)

Tier classification (based on field validation, deployment, and independent confirmation):

Tier 1 – High field validation (≥3 environments, ≥2 years) + commercial deployment (registered cultivar(s) with documented area) + evidence from at least two independent studies (Tier 1 also includes genes with historical commercial success even if currently overcome by pathogen evolution (e.g., *Lr24/Sr24*)).

Tier 2 – Medium field validation (≤2 environments or ≤2 years) + pre−breeding or regional deployment (not yet in widely grown commercial cultivars).

Tier 3 – Low field validation (controlled or single−environment studies without yield data) or a single published report (included as illustrative examples of weak evidence). Tier 3 loci are included as illustrative examples of weak/contradictory evidence; they do not represent validated breeding targets.

Genes that have been commercially deployed in the past but are no longer effective against current pathogen races (e.g., *Lr24/Sr24* due to Ug99-lineage virulence) are still listed as Tier 1 because they represent historically successful introgressions. Their inclusion does not imply current field efficacy.

Genetic unit:

Gene – cloned, function validated

QTL – mapped, gene unknown

Locus – generic (may be gene or QTL, not fully resolved)

Segment – defined alien chromatin, no single gene identified

Field validation:

High – ≥3 environments AND ≥2 years with yield assessment OR widely deployed in commercial cultivars with documented multi−year efficacy

Medium – limited multi−environment trials (≤2 environments or ≤2 years)

Low – controlled or single−environment studies without yield data

Evidence strength (integrates genetic resolution, field validation, and independent confirmation):

Strong – cloned gene (functional validation) + High field validation + ≥2 independent studies.

Moderate – any two of the following: (a) cloned or fine−mapped gene, (b) Medium or High field validation, (c) independent validation (≥2 studies).

Weak – only one of the above, or preliminary/unreplicated data.

Deployment status:

Commercial – registered cultivar(s) with documented area (e.g., *Pm21* > 4M ha)

Regional – used in breeding or released in specific country/region

Pre−breeding – introgressed into wheat but not yet in commercial cultivar

Pre−commercial – advanced pre−breeding with field potential

None – no known deployment

Yield effect: relative to recipient line under the conditions of the study (usually with disease pressure where relevant). Absence of penalty in the absence of disease is not systematically tested.

Linkage drag: qualitative estimate of co−transfer of undesirable alleles (High – severe, Low – minimal, reduced – partially mitigated via recombination).

*Fhb7* vs *Fhb-7EL*: Fhb7 originates from *Th. ponticum* (7el2L, GST gene, HGT origin); *Fhb-7EL* from *Th. elongatum* (7EL). Current evidence does not confirm homology; they may represent distinct loci or alleles. Listed separately to avoid overclaiming.

Evidence strength was evaluated as: Strong – (i) cloned gene + High field validation + ≥2 independent studies, or (ii) locus with diagnostic markers, High field validation, commercial deployment, and confirmed by ≥2 independent studies; Moderate – any two of: cloned/fine−mapped, Medium or High field validation, independent validation; Weak – only one of the above, or preliminary data.

The threshold of ≥3 environments and ≥2 years for Tier 1 was chosen to ensure a minimal assessment of genotype × environment interactions, consistent with widely accepted norms in plant breeding for multi−environment trial design. Commercial deployment is weighted as strong evidence because it represents an independent, large−scale validation that integrates agronomic performance, quality, and farmer acceptance — the ultimate translational milestone. These criteria were developed specifically for this review to assess translational relevance, not genetic novelty. A limitation of this scheme is that historically successful introgressions that are no longer effective against current pathogen races (e.g., *Lr24*/*Sr24)* are retained in Tier 1 to reflect their documented past impact. Conversely, historical resistance loci with proven effectiveness but without evidence of large−scale commercial deployment or modern multi−environment validation (e.g., *Lr29*) were assigned to Tier 3, consistent with the review’s focus on translational outcomes rather than genetic value alone.

### 2.5 Limitations of the review

Our synthesis is qualitative and does not include a formal meta−analysis. Publication bias (overrepresentation of positive results) is likely, as is common in plant science literature, and grey literature from breeding companies was not accessible. The exclusion of *Aegilops* was a deliberate scoping decision to keep the focus on constraints specific to tertiary gene pool transfers; therefore, our findings should not be generalized to introgression from *Aegilops* without further analysis.

## 3 Methodological limitations in trait evaluation

One of the major factors limiting the successful translation of introgressed genetic material from wild Triticeae species into wheat breeding is the methodology used to evaluate target traits. Although numerous studies report positive effects of introgression at physiological and molecular levels, the experimental approaches employed often fail to provide reliable predictions of agronomic performance under field conditions (Dvojković et al., 2023). As a result, a substantial proportion of reported introgressions remain of limited practical value.

### 3.1 The problem of surrogate traits: weak linkage to agronomic performance

A large fraction of studies assessing introgression-derived traits rely on measurements conducted at early developmental stages, typically under controlled conditions such as hydroponic systems, growth chambers, or artificially imposed stress environments. Commonly evaluated parameters include seedling biomass, root and shoot length, chlorophyll content, osmolyte accumulation, antioxidant enzyme activity, and ion homeostasis indicators such as Na^+^/K^+^ ratios under salinity stress (Munns and Tester, 2008; Yuan and Tomita, 2015; Meng et al., 2017; Zeng et al., 2023).

While these traits are often interpreted as proxies for stress tolerance, their relationship with yield and overall plant performance in field conditions is frequently weak or inconsistent. For example, improved ion exclusion or osmotic adjustment at the seedling stage does not necessarily translate into enhanced grain yield under saline soils, where stress dynamics are more complex and vary over the growing season (Meng et al., 2017; Kotula et al., 2024).

This reliance on surrogate traits creates a systematic bias, where introgression lines are classified as «promising» based on laboratory-based indicators that have limited capacity to capture performance under field conditions. Consequently, many reported beneficial effects remain confined to controlled experiments and fail to manifest under realistic agronomic conditions. A structured overview of trait categories and their validation levels is provided in **Supplementary Table 1**.

We do not argue that all physiological or seedling−stage measurements are without merit. Under specific, well−characterized stress scenarios, certain traits—such as canopy temperature depression, carbon isotope discrimination, or spectral reflectance indices—can show useful correlations with yield, particularly when measured with high−throughput platforms and integrated into multi−trait selection indices. However, the predictive value of such traits must be rigorously validated in the target population of environments before they can reliably inform breeding decisions. The central problem identified here is the routine use of unvalidated surrogate traits in introgression studies, which inflates early promise while impeding translatability.

### 3.2 Lack of robust field validation

Another critical limitation is the insufficient validation of introgression lines in field trials. A considerable number of studies report results based on single-location experiments or short-term trials, often without replication across environments. Such designs do not capture genotype × environment interactions, which are particularly important for traits like drought or salinity tolerance (Li et al., 2025; Gholizadeh et al., 2026). In many cases, even when field experiments are conducted, they are limited in scale or lack appropriate controls, making it difficult to assess the true agronomic value of introgressed material. The absence of multi-location and multi-year trials prevents the identification of stable effects and leads to overestimation of trait performance (Kuzmanović et al., 2018).

This issue is further exacerbated by the fact that stress conditions in field environments are inherently heterogeneous. Soil composition, climatic variability, and management practices can significantly influence phenotypic expression, meaning that results obtained in one environment may not be reproducible elsewhere.

### 3.3 Mismatch between levels of analysis: from genes to yield

A fundamental limitation of many introgression studies lies in the disconnect between different levels of biological organization, from gene expression and cellular processes to whole-plant performance and yield. While numerous studies identify candidate genes or physiological mechanisms associated with stress responses, these findings are often not translated into agronomically meaningful outcomes (Lyu et al., 2022; Gholizadeh et al., 2026). At the molecular and cellular levels, introgressed genes frequently affect specific processes such as ion transport, osmotic regulation, or stress signaling. However, these effects do not scale linearly to the level of the whole plant. Yield and agronomic performance are emergent traits resulting from the integration of diverse physiological processes across organs and developmental stages, rather than the direct outcome of single pathways (Tardieu et al., 2018; Luo et al., 2024).

One key reason for this mismatch is the presence of compensatory and regulatory mechanisms at the organismal level. Alterations in one pathway may be buffered by adjustments in others, resulting in limited net effects on growth or reproduction. In addition, resource allocation trade-offs can constrain phenotypic outcomes, particularly for stress-related traits.

**Figure 1:**
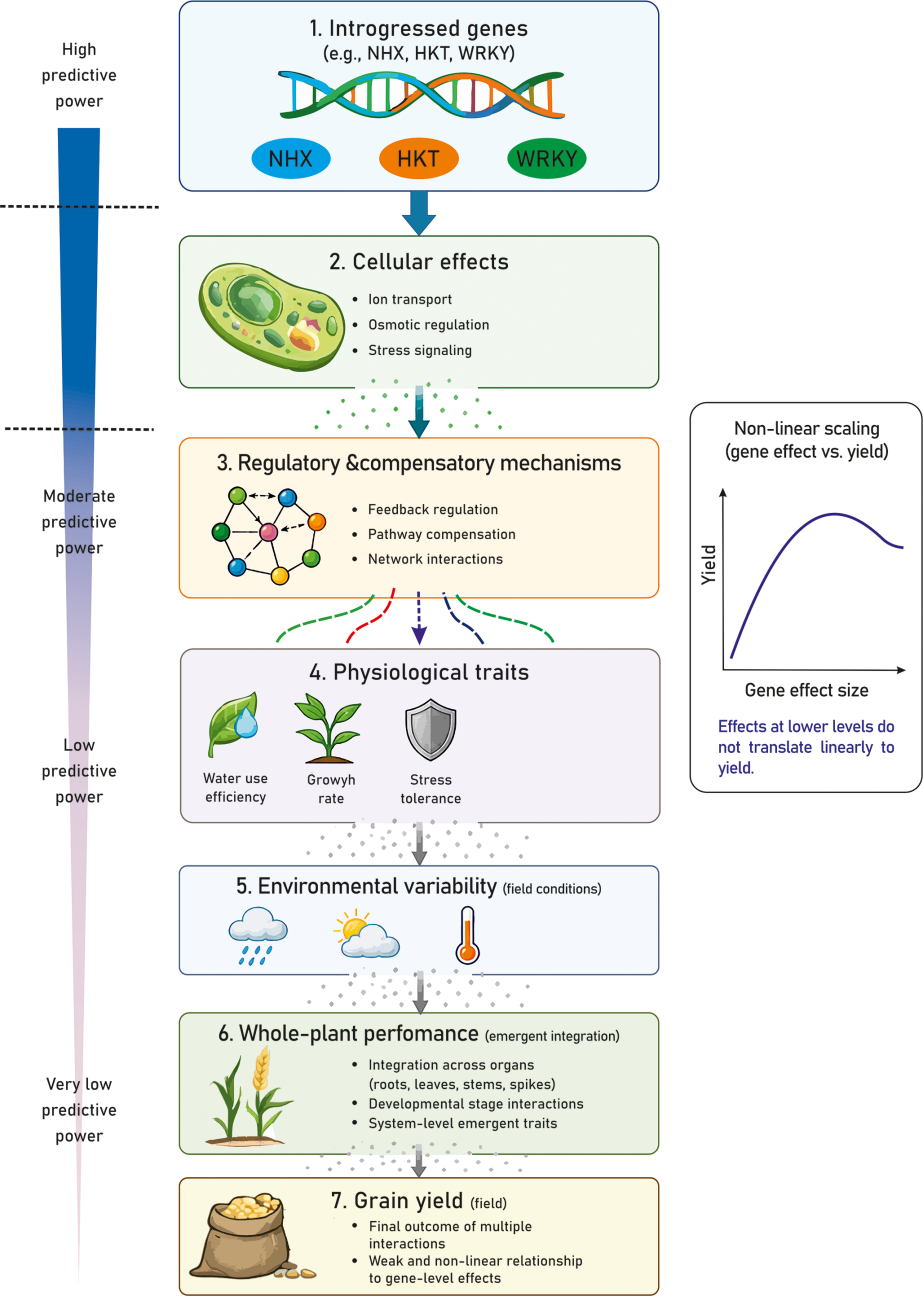
The non−linear translational chain from introgressed genes to grain yield. The diagram illustrates why effects observed at the gene level often fail to translate into agronomic gains. Introgressed genes (e.g., NHX, HKT, WRKY) first influence cellular processes such as ion transport, osmotic adjustment, and stress signalling. At the tissue and organ level, these effects are modulated by regulatory feedback, pathway compensation, and network interactions, which can buffer or dilute the initial signal. Physiological traits (e.g., water−use efficiency, growth rate, stress tolerance) emerge from this integration, but their expression in the field is further altered by environmental variability. Whole−plant performance represents an emergent integration of organ−level responses, developmental stage−dependent interactions, and systemic traits. Grain yield, the final agronomic output, is the result of complex, non−linear interactions among all these levels. The right−hand graph schematically depicts the non−linear scaling of gene effects: beyond a certain point, larger gene−level effects do not produce proportional yield increases.

Scaling effects further contribute to this disconnect. Traits measured at the cellular or seedling level are typically assessed in simplified and highly controlled systems that do not capture the temporal and spatial complexity of agricultural environments (Blum, 2011; Shafqat, 2019). As a result, effects detectable under simplified conditions may be attenuated or lost under realistic agronomic conditions. Together, these factors indicate that the relationship between gene-level effects and yield is inherently non-linear and mediated by multiple layers of biological organization. Failure to account for this complexity contributes to the persistent gap between molecular discoveries and their practical application in wheat breeding. This multi-level disconnect is illustrated in **Figure 1**.

### 3.4 Synthesis: what constitutes reliable evidence?

Taken together, these methodological limitations indicate that a substantial portion of the current literature provides evidence that is often indirect and not directly transferable to breeding decisions. To be considered robust, evidence of successful introgression should meet several criteria: (i) demonstration of trait effects beyond early developmental stages, (ii) validation under agronomically relevant and environmentally variable conditions, preferably across multiple environments, and (iii) a measurable contribution to key agronomic outcomes, particularly yield or yield stability. In the absence of such evidence, the apparent effectiveness of introgressed traits is likely to be inflated by experimental design and evaluation context. The combined effects of surrogate trait reliance, limited field validation, and fragmented levels of analysis contribute to a systematic disconnect between experimental observations and realized performance in breeding programs. Addressing these limitations is essential for improving the reliability and practical relevance of introgression research. The distribution of evidence across traits and validation levels further highlights this imbalance (**Supplementary Table 1**).

## 4 Genetic constraints of introgression

Beyond methodological limitations in trait evaluation, the effectiveness of introgression is fundamentally constrained by genetic factors that govern the expression, stability, and integration of alien chromatin within the wheat genome (Rey et al., 2015; Dong et al., 2020; Kuzmanović et al., 2020; Nyine et al., 2020). These constraints often determine whether an introgressed segment can contribute to agronomic improvement or remains of purely experimental interest.

### 4.1 Polygenic nature of target traits

Many of the traits targeted in introgression studies – such as drought tolerance, salinity tolerance, and yield stability – are inherently polygenic and controlled by complex networks of interacting loci. In such cases, the transfer of a single gene or chromosomal segment from a wild relative is unlikely to produce a consistent and substantial phenotypic effect (Mackay et al., 2009; Bernardo, 2016; Kroupin et al., 2019; Kumar et al., 2022; Gholizadeh et al., 2026).

Moreover, the expression of introgressed alleles is highly dependent on the genetic background of the recipient cultivar. Epistatic interactions between alien and native wheat genes can modify, suppress, or even reverse the expected phenotypic effects. As a result, introgressions that appear beneficial in one genetic background may show reduced or no effect in another (Kuzmanović et al., 2018, 2020; Wang et al., 2018; Pang et al., 2025; Zhang et al., 2025a). This context dependency significantly complicates the use of introgression in breeding programs, where stability and predictability of trait expression are essential (Kuzmanović et al., 2018; Hou et al., 2024).

### 4.2 Linkage drag and segment architecture

A central genetic limitation of introgression is linkage drag-the co-transfer of undesirable genes physically linked to the target locus within the same chromosomal segment (Friebe et al., 1996; Ceoloni et al., 2017; Kruppa et al., 2025). Because recombination between wheat and alien chromatin is often restricted, particularly for distant relatives, introgressed segments frequently encompass multiple linked loci originating from the donor genome (Türkösi et al., 2023).

The size and structural properties of these segments are key determinants of their phenotypic effects (Kroupin et al., 2019). Larger introgressed regions have a higher probability of containing genes affecting diverse biological processes, increasing the likelihood of negative pleiotropic effects and unintended interactions with the recipient genome (Kuzmanović et al., 2018; Hou et al., 2024).

Reducing segment size through recombination or chromosome engineering is therefore a major objective, as it can minimize the co-transfer of deleterious alleles (Hao et al., 2020; Cifuentes et al., 2024). However, this creates a fundamental trade-off, particularly for complex traits. When traits are controlled by multiple loci, reducing introgression size may simultaneously eliminate components of the desired phenotype, limiting overall effectiveness (Zhang et al., 2025a).

Even with *Ph1* suppression (e.g., *ph1b* mutants), recombination between wheat and distant relatives such as *Thinopyrum* remains inherently limited due to structural divergence and low collinearity. Although *ph1b*-induced homoeologous recombination is widely used to facilitate alien introgression, its efficiency remains low and requires large populations and complex crossing schemes (Mandal et al., 2025). Moreover, recent studies demonstrate that recombination breakpoints are often restricted to specific chromosomal regions, resulting in relatively large introgressed segments (e.g., ~48 Mb) even after selection (Wang et al., 2025b). These structural constraints contribute to persistent linkage drag and limit the reduction of alien segments to minimal functional units (Zhang et al., 2025a).

These constraints are especially pronounced for species from the tertiary gene pool, where limited homologous recombination restricts fine-scale introgression. Detailed characteristics of representative introgressed loci, including segment size, chromosomal location, and marker availability, are summarized in **Supplementary Table 2**.

### 4.3 Stability of introgressed segments across generations and environments

The stability of introgressed traits is a critical requirement for their successful deployment in breeding programs. However, even when favorable effects are initially observed, their expression is not always consistently maintained across generations or environments (Chen et al., 2020).

Instability may arise from structural variation, recombination events, or epigenetic regulation affecting introgressed segments (Zhang and Dvořák, 1990; Fu et al., 2013; Rey et al., 2018). In addition, interactions between alien chromatin and the recipient genome can modify gene expression, leading to context-dependent phenotypic outcomes (Antonyuk et al., 2022). For breeding applications, such instability represents a major limitation, as reproducibility across genetic backgrounds and environments is essential. Consequently, stable expression remains a key criterion distinguishing introgressions with practical breeding value from those of primarily experimental relevance (Ceoloni et al., 2017; Perničková et al., 2019).

## 5 Determinants of successful introgression: analysis of successful cases

Despite the limitations outlined above, accumulated evidence demonstrates that the incorporation of alien genetic material into wheat can be successful under specific conditions (**Table 1**). However, such reported cases remain relatively rare and share a consistent set of characteristics that distinguish them from the majority of reported introgressions.

The most consistent outcomes are associated with traits of relatively simple genetic architecture, particularly disease resistance genes. Classical examples include *Lr24* and *Sr24* derived from *Th. ponticum*, as well as the widely utilized 1BL.1RS translocation from *Secale cereale* (Smith, 1996; Plotnikova et al., 2023; Li et al., 2024b; Han et al., 2025). These cases are characterized by large phenotypic effects and clearly defined target traits. However, evidence summarized in **Table 1** indicates that even widely deployed variants may exhibit background-dependent effects and trait-specific trade-offs in agronomic performance. A well-documented example is the 1BL.1RS translocation, where positive effects on grain yield and canopy water status are counterbalanced by deterioration in bread-making quality caused by the loss of wheat *Glu-B3*/*Gli-B1* loci and the introduction of rye secalins, though marker-assisted replacement of those loci can restore dough functionality without sacrificing the yield advantage (Howell et al., 2014; Kaur et al., 2022; Sharma et al., 2024). In contrast, attempts targeting complex traits such as abiotic stress tolerance show substantially lower transferability and tend to be strongly context-dependent. The success of 1BL.1RS, despite its origin from a more distant relative, reflects a rare combination of large phenotypic effects and prolonged field selection rather than a broadly generalizable pattern (Ren et al., 2009; Wang et al., 2025a).

Nevertheless, selected cases indicate that even large chromosomal introgressions can be agronomically viable. For example, wheat-*Th. intermedium* substitution lines carrying chromosome 6Ag^i^2 have been successfully deployed in breeding, combining stable disease resistance with region-specific yield performance (Ivanova et al., 2021). The most striking example is the 6VS·6AL Robertsonian translocation from *Dasypyrum villosum*, deployed on >3.4 million hectares in China: it replaces the wheat *NAM-A1* locus with its functional ortholog *NAM-V1*, which maintains–and may even increase–grain protein content (Zhao et al., 2016). Its lack of a yield penalty reflects a fortuitous absence of deleterious alleles in this particular segment rather than a general property of large introgressions. Similar patterns are observed for targeted substitutions from *Thinopyrum* species (e.g., 5J/5Eb, 3E, 7E), where effects on ion balance and root performance under stress are generally moderate and environmentally dependent (Forster et al., 1988; King et al., 1996, 1997; Zeng et al., 2023; Tounsi et al., 2024).

Synthetic forms and amphiploids represent partially successful systems, where combinations of wheat and wild genomes retain adaptive physiological responses, including altered expression of ion transporters such as *SOS1* and *NHX1* (Gholizadeh et al., 2026). However, these effects are typically background-dependent and rarely translate into stable yield advantages without further selection. A key determinant of success across these cases is the reduction of introgressed segment size. Transitioning from large chromosomal substitutions to smaller recombinant segments reduces linkage drag and improves agronomic performance, forming the basis of modern chromosome engineering approaches and targeted introgression strategies (Yang et al., 2023).

It is instructive to contrast these findings with introgression from the secondary gene pool. In *Aegilops* species, which share at least one homologous genome with wheat, higher homoeologous recombination rates and lower linkage drag have led to notably higher success rates. Well−characterized multipathogen resistance genes such as *Lr34* and *Lr42* have been widely deployed in wheat cultivars worldwide (Abdelrahman et al., 2024). Systematic field evaluation of several hundred *Ae. tauschii* introgression lines revealed that nearly a quarter produced more grain than the parental cultivars—a success rate orders of magnitude higher than that reported for any tertiary gene pool donor (Nyine et al., 2021). Recent population genomic studies in *Ae. tauschii* have identified a rich repertoire of beneficial alleles for disease resistance and yield improvement (Awan et al., 2022; Kou et al., 2023), and the development of speed−introgression platforms now enables the rapid transfer of entire donor genomes into elite wheat backgrounds in as little as two years (Li et al., 2024a). Furthermore, a comprehensive review of U−genome *Aegilops* species highlights the breadth of useful alleles available in this genus, ranging from disease resistance to abiotic stress tolerance (Badaeva et al., 2025). Collectively, these data demonstrate that the translational potential of the secondary gene pool is substantially higher, reinforcing the specificity of the constraints described in this review to more distant tertiary gene pool relatives and underscoring the pivotal role of genomic compatibility and recombination control in successful introgression.

Taken together, these patterns indicate that the success of such transfers is primarily determined by trait architecture and the precision of genomic localization, rather than by the donor species itself (**Table 2**). Apparent differences among genera largely reflect variation in genomic compatibility, recombination potential, and historical breeding focus.

**Table 2:** Comparative breeding relevance of wild Triticeae genera and intergeneric hybrids for wheat introgression.

Genus/system	Field−validated introgressions	Commercial cultivars	Evidence strength	Genetic constraints	Evidence limitations	Overall breeding relevance	Key reference(s)
*Thinopyrum*	High (multiple confirmed)	Yes (multiple loci)	Very strong	Linkage drag; large segments; background−dependent effects	Limited multi−environment yield data for many introgressions	Very high	(Friebe et al., 1996; Qi et al., 2007; Ceoloni et al., 2017)
*Dasypyrum*	High (*Pm21, Pm67, Pm4VL*)	Yes (>40 cultivars with *Pm21*)	Strong	Race specificity; background effects; risk of vulnerability	Primarily disease resistance; limited abiotic validation	Very high (*Pm21*-driven)	(Xing et al., 2018; Zhang et al., 2018, 2021, 2026; Lu et al., 2024; Wei et al., 2024)
*Elymus*	Low-Moderate (FHB QTL candidates)	No	Low-Moderate	Genomic incompatibility; instability; polyphyletic genus	Limited multi−environment trials; no yield data; pre−breeding stage	Low-Moderate	(Cainong et al., 2015; Motsnyi et al., 2024)
*Roegneria*	Very low (experimental reports)	No (genetic resources available)	Low-Moderate (genomic)	Unknown interactions; yield penalties in some reports	Insufficient field validation; only preliminary data	Very low (genomic potential: moderate)	(Song et al., 2023)
*Leymus*	Moderate (BNI lines in CIMMYT trials)	No	Moderate (BNI effect confirmed)	Large introgressed segments; environment−dependent expression	No significant yield effect demonstrated to date	Low–Moderate (trait−validated, yield unconfirmed)	(Subbarao et al., 2021)
*Pseudoroegneria*	None (field)	No	Weak-Moderate (genomic)	No direct introgressions; unknown agronomic traits	Molecular−level characterization only	Very low (genomic relevance: moderate)	(Zhai et al., 2024)
×*Trititrigia* (hybrid system)	Moderate (regional trials)	Yes (regional, Russia)	Moderate	Lower yield potential than elite wheat; limited geographic adaptation	Lower drought tolerance (3.80 t/ha vs 5.09 t/ha in dry zone); data from single country	Moderate (regional importance)	(Lachuga et al., 2023; Shchuklina et al., 2026)

Field−validated introgressions: qualitative assessment based on number of introgressions meeting field validation criteria (see **Table 1** footnote).

Evidence strength:

Very strong – consistent multi−environment evidence across multiple independent studies;

Strong – multiple independent validations;

Moderate – limited independent validation;

Weak – preliminary or inconsistent data.

Overall breeding relevance: qualitative synthesis combining validation level, genetic constraints, and demonstrated deployment success.

×*Trititrigia* is an intergeneric hybrid (wheat × wheatgrass), not a taxonomic genus; included because of its regional breeding importance.

The contrast is quantitative as well as qualitative. Successful introgressions from the secondary gene pool (*Aegilops* species) – such as the multipathogen resistance genes *Lr34* and *Lr42* – typically exhibit higher homoeologous recombination rates and significantly lower linkage drag than tertiary donors (Abdelrahman et al., 2024). Even in a systematic field evaluation of several hundred *Ae. tauschii* introgression lines, nearly a quarter produced more grain than the parental cultivars, a success rate orders of magnitude higher than that reported for any tertiary gene pool donor (Nyine et al., 2021). This reinforces that the limitations highlighted in this review are specific to transfers from more distant relatives.

## 6 Determinants of limited translational success: an integrative synthesis

The limited success of the use of introgressed material in wheat breeding arises from the interaction of methodological and genetic factors that jointly bias evaluation and reduce the likelihood of agronomic impact. Rather than acting independently, these constraints reinforce one another, leading to systematic distortion in the assessment of the value of these materials (Zhang et al., 2025b). A central component of this system is the mismatch between phenotyping approaches and breeding targets. The widespread reliance on early-stage physiological traits introduces an initial selection bias, as these traits have limited predictive value for yield under field conditions (Blum, 2011). Consequently, genotypes identified under controlled conditions often fail to translate into consistent agronomic performance. This limitation is compounded by insufficient multi-environment field validation, which restricts the detection of genotype × environment interactions and reduces reproducibility (Kuzmanović et al., 2018). As a result, many derived lines classified as promising do not demonstrate stable performance in agricultural settings.

Genetic factors further constrain outcomes. The polygenic nature of most target traits reduces the contribution of individual loci and increases dependence on genetic background (Mackay et al., 2009; Bernardo, 2016; Cheng et al., 2025). In addition, linkage within introgressed segments couples beneficial and deleterious alleles, limiting net effects (Friebe et al., 1996; Ceoloni et al., 2017), while instability across generations and environments introduces additional uncertainty (Mohammed et al., 2014; Kuzmanović et al., 2018; Rafiei et al., 2024). Together, these factors create a self-reinforcing system: non-predictive phenotyping generates weak candidates, limited validation fails to eliminate them, and genetic complexity obscures true effects. This results in systematic overestimation at early stages, followed by attrition during later evaluation (**Figure 1**).

**Figure 2:**
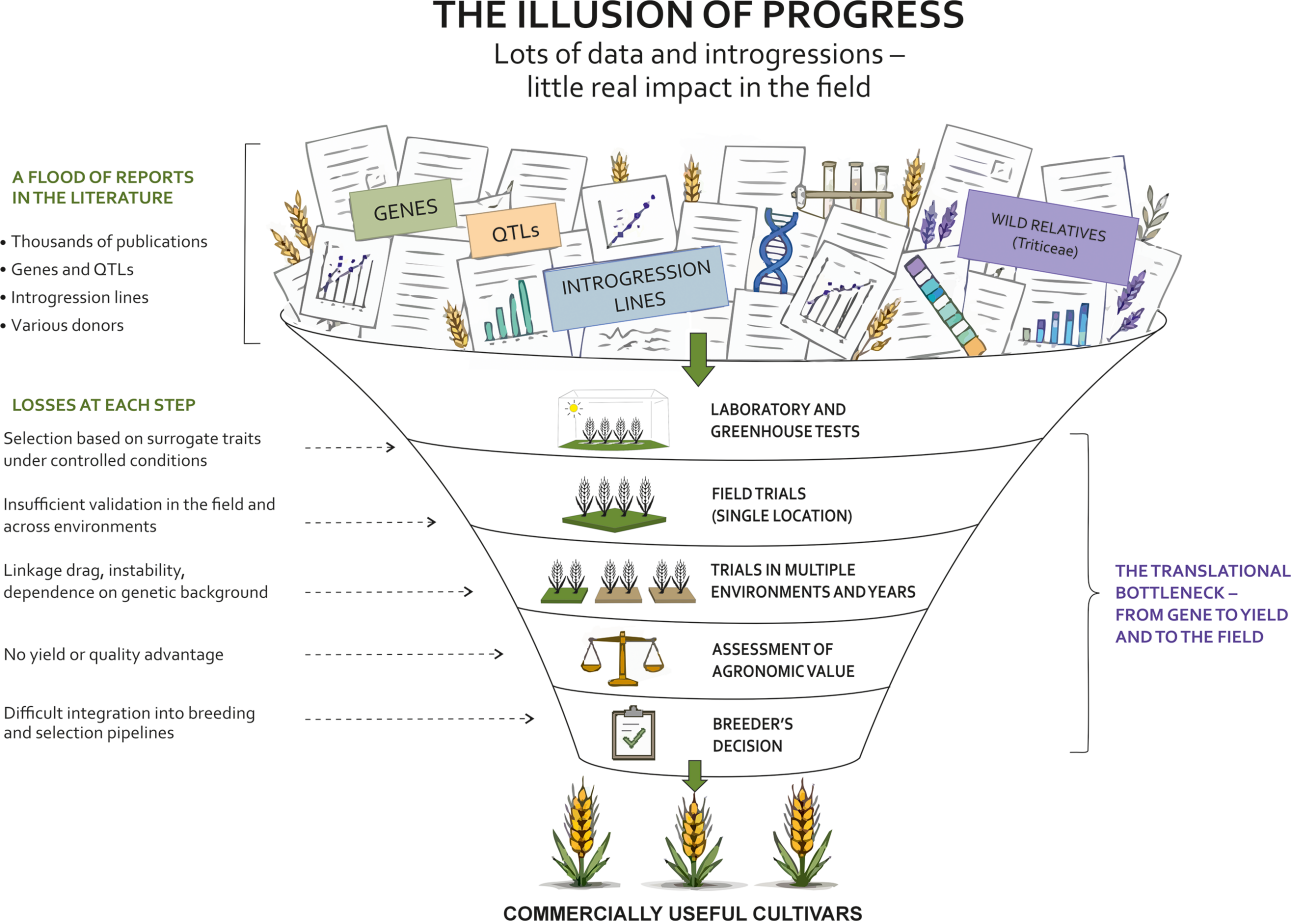
The “illusion of progress” cascade: from wild relatives to commercial cultivars. The infographic tracks the flow of reported introgressions through successive translational filters. The broad top of the funnel represents the abundant literature output: thousands of publications describing genes, QTLs, and introgression lines from various wild Triticeae donors. As material moves through the pipeline, attrition occurs at each stage: laboratory and greenhouse screening often relies on surrogate traits under controlled conditions; subsequent field validation is frequently limited to a single location; multi−environment and multi−year testing, required to assess stability, is rare; rigorous evaluation of agronomic value (yield, quality) is even rarer; and only a handful of cases reach the breeder’s decision point. The right−hand bracket labels this progressive narrowing as the “translational bottleneck — from gene to yield and field.” The few introgressions that survive all filters emerge at the bottom as commercially useful cultivars, illustrating why the volume of published data creates a misleading impression of progress that is not matched by realized breeding outcomes.

This dynamic contributes to an «illusion of progress» – a qualitative synthesis in which publication bias may affect the observed distribution – where the accumulation of candidate genes, QTLs, and introgression lines is interpreted as advancement despite limited gains in yield or agronomic performance (**Figure 2**) (Rafiei et al., 2024; Schreiber et al., 2024; Cheng et al., 2025). Improvements in quality or nutritional traits are often associated with yield trade-offs or remain insufficiently validated (**Supplementary Table 3**). A systematic inventory of 70 unique introgressions from tertiary gene pool Triticeae (**Supplementary Table 4**) quantitatively reinforces this view: only 5 introgressions (7.1%) reached Tier 1 (robust multi-environment validation and commercial deployment), 12 (17.1%) fell into Tier 2 (limited but existing field evidence), while the remaining 53 (75.7%) remained in Tier 3, characterized by low field validation or single reports without independent confirmation. Stratification by donor genus revealed that the genus *Thinopyrum* accounted for all but one Tier 1 introgressions and the vast majority of Tier 2 cases (combined Tier 1 + 2 rate of ~34% within the genus), whereas introgressions from *Dasypyrum*, *Elymus*, *Leymus*, and *Roegneria* only exceptionally progressed beyond Tier 3 (two out of 26 introgressions, both from *Dasypyrum*).

Evidence summarized in **Table 1** further illustrates this imbalance: only a minority of introgressions meet criteria of robust multi-environment validation and clear agronomic impact, whereas most remain supported by partial or context-dependent evidence. Similar patterns have been reported in broader assessments (Dempewolf et al., 2017). Improving outcomes therefore requires coordinated changes in experimental design and evaluation criteria. Phenotyping, genetic analysis, and field validation must be aligned with the objective of stable yield improvement. Without such integration, technological advances alone may be insufficient to overcome existing limitations. An additional constraint is publication bias, whereby positive results are preferentially reported (Fanelli and Glänzel, 2013; Kicinski, 2013). This inflates perceived success and hinders objective evaluation of these strategies. Such a bias would also be mitigated by more balanced reporting, including negative or neutral results, which are currently underrepresented in the literature but essential for understanding the true limitations of introgression. Addressing this requires systematic reporting of multi-environment outcomes, including neutral and negative results, and the adoption of standardized evaluation frameworks emphasizing reproducibility and agronomic relevance.

## 7 Perspectives: how to improve the effectiveness of introgression

The limited translation of introgressed variation into successful breeding applications indicates that incremental improvements in existing approaches are insufficient. Instead, increasing the effectiveness of this approach requires a combination of methodological refinement, genetic strategies, and conceptual shifts in how introgression outcomes are evaluated.

### 7.1 Prioritizing agronomically relevant phenotyping

A primary priority is the transition from surrogate trait-based evaluation to phenotyping directly linked to agronomic performance. This includes systematic multi-environment trials, assessment across developmental stages, and prioritization of yield and yield stability (Blum, 2011; Tardieu et al., 2018). High-throughput phenotyping platforms and remote sensing technologies offer opportunities to connect controlled experiments with field-based evaluation by enabling large-scale, standardized measurements under realistic environments (Araus and Cairns, 2014; Tattaris et al., 2016). However, their effectiveness requires alignment of measured traits with breeding targets rather than physiological proxies.

### 7.2 Reducing linkage drag through precision introgression

Reducing unwanted genomic linkage remains a central objective. Advances in chromosome engineering, induced homoeologous recombination (e.g., manipulation of the *Ph1* locus), and marker-assisted selection have already improved the resolution of introgression (Qi et al., 2007; Ceoloni et al., 2017).

More recently, genome editing technologies, particularly CRISPR/Cas systems, offer the possibility of transferring specific alleles or recreating beneficial variants without introducing large chromosomal segments from donor genomes (Bortesi and Fischer, 2015; Chen et al., 2019; Sun et al., 2024; Ke et al., 2025). While regulatory and technical challenges remain, these approaches have the potential to circumvent some of the inherent limitations of classical introgression. However, genome editing does not resolve key limitations associated with polygenic traits, where multiple loci and their interactions determine phenotypic outcomes. In addition, transformation efficiency and regeneration remain genotype-dependent in wheat, which may limit the practical scalability of these approaches (Ahmar et al., 2023). Recent advances, such as the use of a GRF4-GIF1 fusion protein to boost regeneration, have increased transformation efficiency up to 60-fold and enabled direct genome editing in several elite spring wheat cultivars (Biswal et al., 2023), partially addressing this bottleneck. However, the improvement remains genotype−dependent, and its broader applicability across diverse wheat genetic backgrounds is yet to be demonstrated.

### 7.3 Integrating introgression with genomic selection

Given the polygenic nature of many target traits, introgression strategies should be integrated with genomic selection frameworks. Rather than focusing on individual genes, genomic selection enables the simultaneous evaluation of cumulative effects across multiple loci, including introgressed segments (Crossa et al., 2017; Kumar et al., 2024; Sun et al., 2024). This approach is particularly relevant for complex traits such as yield, drought tolerance, and yield stability, where individual loci typically have small and context-dependent effects. Incorporating introgression lines into genomic prediction models allows both additive and non-additive effects to be captured within a unified framework, improving the efficiency of selection under realistic breeding conditions. Empirical studies in wheat demonstrate that genomic selection can enhance prediction accuracy and accelerate genetic gain for agronomically important traits, supporting its integration with introgression-based strategies (Lozada et al., 2019; Kumar et al., 2024; Sun et al., 2024).

### 7.4 Functional validation beyond candidate genes

A shift is needed from candidate gene identification to rigorous functional validation. This includes demonstrating the effect of introgressed genes on whole-plant performance and yield in breeding-relevant environments, as well as confirming their stability across genetic backgrounds. Approaches such as expression quantitative trait locus (eQTL) mapping, transcriptomics, and systems biology can help elucidate the regulatory networks underlying introgressed traits (Kliebenstein, 2009; Jin et al., 2021; Zhang et al., 2025b). However, these data must be linked to phenotypic outcomes to be meaningful for breeding.

### 7.5 Targeting compatible genetic backgrounds

The success of introgression is strongly influenced by genomic compatibility between donor and recipient genomes. Therefore, selecting appropriate recipient backgrounds and testing introgressions across multiple genotypes should become standard practice (Hao et al., 2020; Boehm and Cai, 2024). Introgressions from more closely related species are generally easier to stabilize and may serve as initial targets, while more distant introgressions may require additional strategies such as pre-breeding or bridge crosses.

### 7.6 From «more introgressions» to «better introgressions»

A fundamental conceptual shift is required: progress should not be evaluated by the number of identified genes or introgression lines, but by their demonstrated agronomic impact (Singh et al., 2021). This implies prioritizing introgressions that meet strict criteria of stability, reproducibility, and agronomic relevance. Such a shift would also promote more balanced reporting, including negative or neutral results, which are currently underrepresented in the literature but essential for understanding the true limitations of introgression.

## 8 Conclusions

The use of wild Triticeae species for the introgressive improvement of bread wheat remains a promising but still limited strategy in terms of practical breeding impact. Despite extensive research and the identification of numerous genes and introgression lines, only a small fraction has been successfully translated into agronomically valuable cultivars. It must be stressed that these findings apply primarily to tertiary gene pool transfers. Introgression from the secondary gene pool (*Aegilops* spp.) has a substantially higher success rate, consistent with greater genomic compatibility, and our conclusions should not be extrapolated to those species without further evidence. This review shows that the primary barriers to success arise not from a lack of genetic variation, but from interacting methodological and genetic constraints that limit the reliable identification and deployment of effective introgressions. These constraints reinforce one another, leading to a possible systematic overestimation of reported effects and a clear imbalance between «promising» findings and realized breeding outcomes.

Introgressions that achieve practical success share a consistent set of features, including association with major-effect genes, minimal linkage drag, stable expression across environments, and clear agronomic relevance. In contrast, introgressions targeting highly polygenic traits or involving large chromosomal segments from distant relatives are substantially less likely to succeed.

Improving outcomes will require both technical and conceptual advances, including better alignment between phenotyping, genetic analysis, and field validation, as well as a shift toward evaluating introgression based on demonstrated agronomic impact rather than molecular characterization alone. A practical constraint is the time and resource intensity of breeding programs, as multi-environment validation over multiple years (often 8–12 years) limits the scale at which introgression lines can be rigorously tested (Tester and Langridge, 2010; Iwańska et al., 2025). This duration also reflects economic constraints, as long breeding cycles and extensive field testing limit the scalability of introgression programs in commercial settings. Overall, the future success of introgression will depend on a more critical and application-oriented framework that prioritizes reproducibility, stability, and measurable gains in agronomic performance.
