# Supplementary material for: The illusion of progress: why most introgressions from wild Triticeae fail to improve wheat agronomically
Source: Front Plant Sci. 2026 Jul 3;17:1886864. doi: 10.3389/fpls.2026.1886864 (PMC13375625; doi:10.3389/fpls.2026.1886864)
Supplement: Supplementary file 1 [file Table1.docx]

Supplementary Material

**Supplementary Table S1. Trait categories, validation levels, and translational status of wild Triticeae introgressions in wheat**

| Trait category | Trait level | Specific trait | Donor genus | Donor species (examples) | Validation level | Translation level | Key references |
| --- | --- | --- | --- | --- | --- | --- | --- |
| Salinity tolerance | Whole-plant | Yield under salinity (amphiploids, saline soils, one location) | *Thinopyrum* | *Th. bessarabicum* | Medium | Breeding | (Khokhar et al., 2020) |
| Salinity tolerance | Chromosome-level | Salt tolerance via 5E^b^ introgression | *Thinopyrum* | *Th. bessarabicum* | Low | Pre-breeding | (King et al., 1996, 1997) |
| Salinity tolerance | Chromosome-level | Seedling salt tolerance (disomic substitution line 3E(3D)) | *Thinopyrum* | *Th. elongatum* | Low | Pre-breeding | (Zeng et al., 2023) |
| Salinity tolerance | Gene-level | Salt tolerance via MYB transcription factor TtMYB1 | *Thinopyrum* | *Th. elongatum* | Low | Molecular (candidate) | (Mu et al., 2024; Tian et al., 2024) |
| Salinity tolerance | Whole-plant | Salt tolerance and post‑stress recovery in *Tritipyrum* amphiploids; candidate *LEA* gene *TtLEA2‑1* | *Thinopyrum* | *Th. elongatum* | Low | Pre-breeding | (Yang et al., 2021; Peng et al., 2022) |
| Salinity tolerance | Gene-level | Candidate salt‑tolerance genes (WRKY, NAC, bHLH families) identified in *Tritipyrum* | *Thinopyrum* | *Th. elongatum* | Low | Molecular (candidate) | (Li et al., 2022b, Li et al., 2025c; Liu et al., 2024) |
| Salinity tolerance | Chromosome-level | Seedling salt tolerance (disomic addition lines) | *Dasypyrum* | *D. villosum* | Low | Experimental | (Han et al., 2023) |
| Salinity tolerance | Chromosome-level | Disomic addition lines of individual chromosomes or arms of these species were used in the background of Chinese Spring wheat | *Dasypyrum* | *D. villosum* | Low | Pre-breeding | (Zhong and Dvořák, 1995) |
| Drought tolerance | Chromosome-level | Adaptation to drought with the help of an improved root system in the translocation line of wheat T7DL•7Е(Ag) | *Thinopyrum* | *Th. elongatum* | Medium | Pre-breeding | (Placido et al., 2013) |
| Drought tolerance | Whole-plant | Drought response traits in amphiploids (controlled conditions) | *Thinopyrum* | *Th. bessarabicum* | Low | Experimental | (Khokhar et al., 2020) |
| Drought tolerance | Whole-plant | Flowering-stage drought tolerance (chromosome 3St substitution line) | *Thinopyrum* | *Th. ponticum* | Low | Physiological | (Türkösi et al., 2025) |
| Drought tolerance | Chromosome-level | Drought tolerance during flowering in translocated wheat line (T4StS•1JᵛˢS) | *Thinopyrum* | *Th. ponticum*  *Th. intermedium* | High | Pre-breeding | (Kruppa et al., 2025) |
| Drought tolerance | Chromosome-level | Leaf pubescence associated with drought adaptation; donor chromosome 4Th carrying the newly identified pubescence gene *Hl1th* | *Thinopyrum* | *Th. ponticum* | Low | Pre-breeding | (Simonov et al., 2024) |
| Drought tolerance | Chromosome-level | Root system enhancement (greenhouse/lysimeter; translocation carries segments from *Ae. speltoides* and *D. villosum*) | *Dasypyrum Aegilops* | *D. villosum*  *Ae. speltoides* | Low | Experimental | (Djanaguiraman et al., 2019) |
| Perenniality | Whole-plant | Polycarpic growth habit (chromosome addition) | *Thinopyrum* | *Th. elongatum* | Low | Experimental | (Lammer, 2004) |
| Stem rust | Gene-level | *Sr24, Sr25, Sr26* (resistance genes) | *Thinopyrum* | *Th. ponticum* | High | Breeding (commercial) | (Knott, 1961; Plotnikova et al., 2023a; Li et al., 2024) |
| Stem rust | Gene-level | *Sr43* (unusual protein kinase; cloned; transgenic validation) | *Thinopyrum* | *Th. ponticum* | Medium | Pre‑breeding | (Niu et al., 2014; Yu et al., 2023; Zhao et al., 2025) |
| Stem rust | Chromosome-level | *Sr68* as part of the translocation | *Thinopyrum* | *Th. junceum* | Low | Pre‑breeding | (Mandal et al., 2025) |
| Stem rust | Gene-level | *Sr52* (mapped, temperature‑sensitive) | *Dasypyrum* | *D. villosum* | Low | Pre‑breeding | (Li et al., 2019) |
| Yellow rust | Gene-level | *Yr4EL* (mapped to 4EL) | *Thinopyrum* | *Th. elongatum* | Medium | Pre‑breeding | (Gong et al., 2024, 2026) |
| Yellow rust | Gene-level | *Yr69* (mapped to 2AS) | *Thinopyrum* | *Th. ponticum* | Low | Pre‑breeding | (Hou et al., 2016) |
| Yellow rust | Gene-level | *YrTp1, YrTp2* (mapped loci, no subsequent validation reported) | *Thinopyrum* | *Th. ponticum* | Low | Pre‑breeding | (Yin et al., 2006) |
| Yellow rust | Chromosome-level | Translocated line (T1BL•1BS-3A) | *Thinopyrum* | *Th. intermedium* | Low | Pre‑breeding | (Zheng et al., 2020) |
| Yellow rust | Chromosome-level | Translocated line (T3AL-7StS•7StL) | *Thinopyrum* | *Th. intermedium* | Low | Pre‑breeding | (Li et al., 2025b) |
| Yellow rust | Chromosome-level | Resistance located on chromosome arm 4J^S^L | *Thinopyrum* | *Th. intermedium* | Medium | Pre‑breeding | (Li et al., 2022a) |
| Yellow rust | Gene-level | *YrT14* (mapped to 7J or 7J^s^) | *Thinopyrum* | *Th. intermedium* | Medium | Pre‑breeding | (Guo et al., 2023a) |
| Yellow rust | Chromosome-level | The short arm of chromosome 2St | *Thinopyrum* | *Th. intermedium* | Medium | Pre‑breeding | (Jiang et al., 2025) |
| Yellow rust | Gene-level | *YrCD-3* (mapped to 3V) | *Dasypyrum* | *D. villosum* | Medium | Pre‑breeding | (Zhang et al., 2022a) |
| Yellow rust | Gene-level | *Yr7VS* (mapped to 7V) | *Dasypyrum* | *D. villosum* | Medium | Pre‑breeding | (Hou et al., 2024b) |
| Yellow rust | Chromosome-level | Resistance in a disomic additional line with chromosome 7Vb | *Dasypyrum* | *D. breviaristatum* | Low | Experimental | (Li et al., 2016) |
| Powdery mildew + yellow rust | Chromosome-level | T5DL•5V#5S introgression line and related D/V substitution | *Dasypyrum* | *D. villosum* | Medium | Pre‑breeding | (Zhang et al., 2022b) |
| Fusarium head blight + yellow rust | Chromosome-level | Wheat-*E. repens* chromosomal translocation lines carrying resistance to both FHB and stripe rust (3St) | *Elymus* | *E. repens* | Medium | Pre‑breeding | (Gong et al., 2019) |
| Fusarium head blight + yellow rust | Chromosome-level | Substitution lines 2Ns(2D), 3Ns(3D), 4Ns(4D) | *Leymus* | *L. mollis* | Medium | Pre‑breeding | (Zhao et al., 2019; Du et al., 2022; Feng et al., 2022) |
| Fusarium head blight + yellow rust | Chromosome-level | 7Ns alien chromosome | *Leymus* | *L. mollis* | Medium | Experimental | (Du et al., 2026) |
| Powdery mildew + yellow rust | Chromosome-level | Wheat-*Th. ponticum* 1J^s^(1D) disomic substitution line | *Thinopyrum* | *Th. ponticum* | Medium | Pre‑breeding | (Wang et al., 2020b) |
| Powdery mildew + leaf rust resistance | Chromosome-level | wheat-*Th. ponticum* disomic substitution line DS5Ag(5D) | *Thinopyrum* | *Th. ponticum* | Medium | Pre‑breeding | (Zhang et al., 2024) |
| Powdery mildew | Chromosome-level | wheat-*Th. ponticum* disomic addition line (7St) | *Thinopyrum* | *Th. ponticum* | Medium | Pre‑breeding | (Cheng et al., 2025) |
| Powdery mildew | Chromosome-level | wheat-*Th.ponticum* derivative lines (putative/temporarily designated gene *PmSN0293*) | *Thinopyrum* | *Th. ponticum* | Medium | Pre‑breeding | (He et al., 2017; Li et al., 2022c) |
| Powdery mildew | Gene-level | *Pm21* (CC‑NBS‑LRR, widely deployed in commercial cultivars) | *Dasypyrum* | *D. villosum* | High | Breeding (commercial) | (Xing et al., 2018; Ye et al., 2019) |
| Powdery mildew | Gene-level | *Pm55* (developmental‑stage specific; cloned alleles) | *Dasypyrum* | *D. villosum* | Medium | Pre‑breeding | (Lu et al., 2024) |
| Powdery mildew | Chromosome-level | T2BS•2VL#5 Robertsonian translocation | *Dasypyrum* | *D. villosum* | Medium | Pre‑breeding | (Zhang et al., 2018) |
| Powdery mildew | Chromosome-level | wheat-*R. ciliaris* disomic addition line (resistance locus *PmRc1* on chromosome 1S^c^) | *Roegneria* | *R. ciliaris* | Medium | Pre‑breeding | (Cheng et al., 2024) |
| Powdery mildew | Chromosome-level | 3Ns(3D) substitution effects on agronomic traits | *Leymus* | *L. mollis* | Medium | Experimental | (Li et al., 2021) |
| Leaf rust | Gene-level | *Lr19* (linked to *PSY‑E1*; linkage broken in some lines but not widely deployed) | *Thinopyrum* | *Th. ponticum* | High | Pre‑breeding | (Knott, 1968; Kuzmanović et al., 2018; Xu et al., 2023) |
| Leaf rust | Gene-level | *Lr24* (linked to *Sr24*; effective for ~30 years in India) | *Thinopyrum* | *Th. ponticum* | High | Breeding | (Smith et al., 1968; Sears, 1973; Sivasamy et al., 2022; Plotnikova et al., 2023a; Li et al., 2024) |
| Leaf rust | Chromosome-level | A novel resistance factor on chromosome 7J^s^ | *Thinopyrum* | *Th. intermedium* | Medium | Pre‑breeding | (Yang et al., 2023) |
| Leaf rust | Chromosome-level | Translocated line (T3DS-3AS.3AL-7StS) | *Thinopyrum* | *Th. intermedium* | Low | Pre-breeding | (Li et al., 2025b) |
| Leaf rust | Chromosome-level | Wheat-*E. sibiricus* addition lines (3St) | *Elymus* | *E. sibiricus* | Medium | Pre-breeding | (Motsnyi et al., 2024) |
| Leaf rust + stripe rust | Chromosome-level | Wheat-*E. trachycaulus* translocation line (T1H^t^S•1BL) | *Elymus* | *E. trachycaulus* | Low | Pre-breeding | (Friebe et al., 2005; Wang et al., 2025) |
| Fusarium head blight | Gene-level | *Fhb7* (GST-like gene; linkage drag partially mitigated in specific alleles; not yet commercially deployed) | *Thinopyrum* | *Th. ponticum* | Medium | Pre‑commercial | (Guo et al., 2015; Zhang et al., 2022c; Zhao et al., 2024) |
| Fusarium head blight | Gene-level | *Fhb6* (mapped; transferred to wheat; effective in greenhouse) | *Elymus* | *E. tsukushiensis* | Medium | Pre‑breeding | (Cainong et al., 2015) |
| Fusarium head blight | Chromosome-level | *FhbRc1* from 1S^c^ and *FhbRc2* from 3S^c^L | *Roegneria* | *R. ciliaris* | Medium | Pre‑breeding | FhbRc1  (Song et al., 2023)  FhbRc2  (Song et al., 2024) |
| Stripe rust + Fusarium head blight | Chromosome-level | Stripe rust + Fusarium head blight resistance via Ns chromosomes (2Ns, 3Ns, 4Ns) | *Leymus* | *L. mollis* | Medium | Pre‑breeding | (Zhao et al., 2019; Du et al., 2022; Feng et al., 2022) |
| Viruses (BYDV) | Gene-level | *Bdv2* | *Thinopyrum* | *Th. intermedium* | Medium | Breeding (commercial) | (Hohmann et al., 1996; Qonaah et al., 2026) |
| Viruses (WSMV) | Gene-level | *Wsm1* (effective in field trials; temperature‑sensitive) | *Thinopyrum* | *Th. intermedium* | High | Breeding (commercial) | (Wells et al., 1982; Sharp et al., 2002; Guttieri et al., 2023) |
| Septoria blotch | Whole-plant | Wheat introgressive lines carrying *Th. ponticum* genetic material | *Thinopyrum* | *Th. ponticum* | Medium | Breeding (regional) | (Plotnikova et al., 2023b) |
| Disease complex | Chromosome-level | *6Ag^i^2* (complex resistance to fungal diseases; neutral yield effect) | *Thinopyrum* | *Th. intermedium* | Medium | Breeding (regional) | (Salina et al., 2015; Ivanova et al., 2021) |
| Sharp eyespot | Chromosome-level | Wheat-*D. villosum* disomic substitution lines (2V) | *Dasypyrum* | *D. villosum* | Medium | Pre-breeding | (Liu et al., 2023) |
| Cyst nematode | Gene-level | *CreV* (6V) | *Dasypyrum* | *D. villosum* | Low | Pre‑breeding | (Zhang et al., 2016) |

**Supplementary Table S2. Detailed characterization of introgressed wild Triticeae loci in bread wheat: segment size, marker type**

| Gene / locus | Trait | Donor species | Chromosomal location | Segment size (typical/engineered) | Marker type | Main limitation(s) | Key reference(s) |
| --- | --- | --- | --- | --- | --- | --- | --- |
| *Lr19* | Leaf rust | *Th. ponticum* | 7EL translocated to 7DL (e.g., T7DS•7DL-7EL#1) | Large (reduced recombinants available) | Diagnostic (FISH, GISH, STS, SSR, EST) | Yellow pigment linkage (*PSY-E1*); yield penalty in some backgrounds | (Knott, 1968; Xu et al., 2023) |
| *Lr55* | Leaf rust | *E. trachycaulus* | T1HtS•1BL (Robertsonian) | Large (whole arm) | Linked (SSR/microsatellite, DArT markers, FISH) | Limited validation; no commercial deployment | (Friebe et al., 2005; Pietrusińska and Tyrka, 2021; Wang et al., 2025) |
| *Lr24 /Sr24* | Leaf/stem rust | *Th. ponticum* | 3AgL (bin FL 0.85-1.00; breakpoints vary) | Large (variable, e.g., FL 0.85–1.00) | Diagnostic (FISH, GISH, PLUG) | Linkage drag (red grain, RAg in bin FL 0.70–0.85); *Sr24* ineffective against TTKST | (Smith et al., 1968; Plotnikova et al., 2023a; Li et al., 2024) |
| *Sr25* | Stem rust | *Th. ponticum* | T7DS•7DL-7Ae#1L | Large (reduced variants reported) | Co-dominant STS-marker (BF145935) | Strong linkage drag (yellow pigment, PSY-E1); yield penalty reported in some backgrounds | (Liu et al., 2010; Plotnikova et al., 2023a; GrainGenes,  n.d.)) |
| *Sr26* | Stem rust | *Th. ponticum* | T6AS•6AL-6Ae#1L (distal) | Large (reduced recombinants available) | Diagnostic (RFLP, isozyme or PCR-based markers, GISH | Initial ~9% yield penalty (mitigated in recombinants); background‑dependent | (Dundas et al., 2007, 2015) |
| *Sr43* | Stem rust | *Thinopyrum spp. (introgressed from Th. ponticum; gene cloned from Th. elongatum)* | 7el2L (original large segment; engineered recombinants on 7DL) | Large (original, near‑arm replacement), small (engineered, ~13-17%) | Functional (cloned), plus linked SSR, FISH, GISH | Not yet deployed; original large segment with linkage drag; yellow pigment linkage reduced but not fully eliminated | (Niu et al., 2014; Yu et al., 2023) |
| *Sr52* | Stem rust | *D. villosum* | 6V#3L (T6AS•6V#3L, bin FL 0.92‑1.00) | Small (sub‑arm in recombinants) | Diagnostic (STS, EST markers, FISH, GISH) | Temperature‑sensitive (effective at 16°C, ineffective at 28°C) | (Li et al., 2019) |
| *Yr69* | Yellow rust | *Th. ponticum* | 2AS (bin 2AS-0.78-1.00) | Unknown | Linked (SSR/EST-SSR/STS) | QTL not cloned; no multi‑environment validation | (Hou et al., 2016) |
| *YrTp1 / YrTp2* | Yellow rust | *Th. ponticum* | 2BS (dominant) / 7BS (recessive) | Unknown | Linked (SSR) | Single‑study loci; no independent validation | (Yin et al., 2006) |
| *Fhb7* | FHB | *Th. ponticum* | 7EL-derived segment introgressed into 7D (variable lines) | Large (truncated recombinants available; allele Fhb7The2 with reduced segment and broken PSY‑E2 linkage) | Diagnostic (SSR/STS/EST-derived/conserved PCR markers, DArT for mapping, FISH/GISH) | Linked to *PSY-E2* (broken in specific alleles; Li et al., 2023); HGT from fungus; background‑dependent effects; validated in NILs without yield penalty | (Guo et al., 2015, 2023c; Wang et al., 2020a; Zhang et al., 2022c; Li et al., 2023) |
| *Fhb6* | FHB | *E. tsukushiensis* | 1Ets#1S subterminal (replacing 1AS) | Medium (sub‑arm) | Linked (EST–STS/CAPS + KASP/KASPar SNP markers, FISH/GISH) | Instability in wheat background; limited validation | (Cainong et al., 2015) |
| *Pm21* | Powdery mildew | *D. villosum* | T6VS•6AL (Robertsonian) | Large (whole arm) | Diagnostic (PCR-markers, FISH/GISH) | Race specificity; widely deployed in China (>4M ha) | (Xing et al., 2018; Ye et al., 2019) |
| *Pm55* | Powdery mildew | *D. villosum* | 5VS FL 0.60-0.80 (T5VS•5AL, T5VS•5DL) | Small (sub‑arm) | Diagnostic (InDel + EST-STS + CNL2/gene-specific PCR markers, FISH, GISH) | Developmental-stage & tissue‑specific resistance | (Lu et al., 2024) |
| *CreV* | Cyst nematode | *D. villosum* | 6V#4L FL 0.80-1.00 (T6V#4L•6AS, T6V#4L-4BL•4BS, T6V#4L•6V#4S-7BS and DT6V#4L.) | Small (sub‑arm) | Diagnostic (STS, EST-PCR markers, FISH, GISH) | Greenhouse validation only; no field trial data | (Zhang et al., 2016) |
| *Bdv2* | BYDV | *Th. Intermedium* | T7DL•7Ai#1L (distal) | Large | Diagnostic (RAPD-derived SCAR, EST-STS/EST-SSCP PCR, RFLP/SSR-linked markers, FISH, GISH) | Large segment; widely used in Europe (RGT Wolverine) | (Larkin et al., 2002; Gao et al., 2009; Silva et al., 2022; Qonaah et al., 2026) |
| *Wsm1* | WSMV | *Th. Intermedium* | T4DL•4Ai#2S | Large (whole arm; shortened rec213 variant available) | Diagnostic (STS, KASP) | Strong temperature sensitivity (effective ≤18°C, ineffective ≥27°C); limited field performance in warm environments; deployed in Mace, RonL | (Wells et al., 1982; Guttieri et al., 2023) |

**Supplementary Table S3. Reported effects of wild Triticeae introgressions on grain quality, nutritional traits, and yield trade‑offs in bread wheat: a pre‑breeding reference**

| Donor species | Trait | Genetic basis | Effect (quantitative where available) | Context / trade‑off | Field validation | Translation level | Deployment status | Key reference(s) |
| --- | --- | --- | --- | --- | --- | --- | --- | --- |
| *Th. elongatum* | Gluten strength, protein content | HMW‑GS (1Ey) | Protein +3.76-5.11%; wet gluten +6.55-8.73%; bread volume +33.8-246.5 mL | Background‑dependent (effects vary by substitution type, Glu‑D1 background); no universal yield penalty established | Low (controlled conditions, no multi‑environment field yield data) | Pre‑breeding | None | (Dai et al., 2023) |
| *Th. elongatum* | Carotenoids | *PsyE1* (Phytoene Synthase) | Increased carotenoid content (qualitative) | Not demonstrated in wheat background (candidate gene, in silico + PCR only) | Very low (in silico / molecular only) | Molecular (candidate) | None | (Padhy et al., 2023) |
| *Th. ponticum* | Carotenoids | *Lr19*‑linked alien segment (*PsyE1* locus) | Increased yellow pigment content | Yield loss observed in some environments (e.g., Japan: decreased grain number/spike, test weight, TKW); penalty not universal | Medium (multi‑year field trials, limited environments) | Breeding | Regional / historical | (Kato et al., 2025) |
| *Th. ponticum* | Anthocyanin | 4Th substitution (blue aleurone, Ba‑like) | Elevated total anthocyanin content (within experimental set) | Decreased grain weight/main spike, spike number/plant, 1000‑grain weight | Low (single‑environment field trial) | Experimental | None | (Gordeeva et al., 2022) |
| *Th. intermedium* | Grain quality, yield | 4J^r^/St substitution (DS4B/4J^r^/St, DS4D/4J^r^/St) | Improved yield‑related traits relative to parental line (single‑location evidence); higher protein content | Not reported / not quantified within the study | Medium (3‑year field trials, single location) | Pre‑breeding | None | (Wu et al., 2025) |
| *D. breviaristatum* | Yield components | 6V^b^S chromatin introgression | Increased tillering observed in cytogenetic lines (not agronomically validated) | Not quantified | Low (cytogenetic characterization only) | Experimental | None | (Jiang et al., 2022) |
| *R. ciliaris* | Spikelet number | 1ScS•1AL translocation | More spikelets per spike (+15-20% in specific line) | Estimate likely unstable across environments (single‑study QTL‑like effect) | Medium (field trials with limited replication) | Pre‑breeding | None | (Cheng et al., 2024) |
| *L. mollis* | Grain quality (protein, sedimentation) | 1Ns(1D) substitution | Higher protein content and sedimentation value; altered storage protein profile | Not reported / not quantified | Low (single‑environment evaluation) | Pre‑breeding | None | (Li et al., 2025a) |
| *Th. intermedium* and/or *Th. ponticum* | Protein content, grain quality | Multiple (wheat × wheatgrass hybrid ×*Trititrigia*) | Protein >19%; gluten 33.34%; reported yield 4.28 t/ha (context‑dependent) | Lower yield vs. winter wheat (5.85 t/ha vs. 4.28 t/ha); comparability limited by different growing conditions | Medium (multi‑year regional trials) | Breeding | Regional (Russia) | (Lachuga et al., 2023) |
| *Th. intermedium* and/or *Th. ponticum* | Anthocyanin + baking quality | Blue aleurone (Ba introgression) via wheat × wheatgrass hybrid ×*Trititrigia* | Stable grain yield 3.0-3.2 t/ha; high‑quality grain; good baking properties | Lower yield vs. elite wheat | Medium (field trials, single region) | Pre‑breeding | None (experimental line) | (Shchuklina et al., 2026) |

**Supplementary Table S4. Complete inventory of reviewed introgressions from wild Triticeae into bread wheat with tier classification, evidence levels, and deployment status.**

| Gene/Locus/Introgression | | Trait | Genus | Donor species | Tier | Field validiation | Evidence | Deployment | Key limitation / Reason for Tier | Key references |
| --- | --- | --- | --- | --- | --- | --- | --- | --- | --- | --- |
| Tier 1 (5 introgressions, 7.1%) | | | | | | | | | | |
| *Lr24 / Sr24* | | Leaf/stem rust | *Thinopyrum* | *Th. ponticum* | 1 | High | Strong | Commercial | Historical success; *Sr24* virulence emerged | (Smith et al., 1968; Plotnikova et al., 2023a; Li et al., 2024) |
| *Lr19* | | Leaf rust | *Thinopyrum* | *Th. ponticum* | 1 | High | Strong | Commercial | Linkage drag with yellow pigment (*Psy-E1*) | (Knott, 1968; Xu et al., 2023) |
| *Sr26* | | Stem rust | *Thinopyrum* | *Th. ponticum* | 1 | High | Strong | Commercial | Initial 9% yield penalty; mitigated in recombinants | (Dundas et al., 2007, 2015) |
| *Pm21* | | Powdery mildew | *Dasypyrum* | *D. villosum* | 1 | High | Strong | Commercial | Race specificity risk | (Xing et al., 2018; Ye et al., 2019) |
| *Wsm1* | | WSMV | *Thinopyrum* | *Th. intermedium* | 1 | High | Strong | Commercial | Temperature-sensitive; rec213 variant improved | (Wells et al., 1982; Sharp et al., 2002; Guttieri et al., 2023) |
| Tier 2 (12 introgressions, 17.1%) | | | | | | | | | | |
| *Sr25* | | Stem rust | *Thinopyrum* | *Th. ponticum* | 2 | Medium | Moderate | Pre-breeding | Yellow pigment linkage; no independent valid. | (Liu et al., 2010; Plotnikova et al., 2023a) |
| *Sr43* | | Stem rust | *Thinopyrum* | *Th. ponticum/elongatum* | 2 | Medium | Moderate | Pre-breeding | Large original segment; not yet deployed | (Niu et al., 2014; Yu et al., 2023) |
| *Fhb7* | | FHB | *Thinopyrum* | *Th. ponticum* | 2 | Medium | Moderate | Pre-commercial | Linkage drag; HGT origin | (Guo et al., 2015; Wang et al., 2020a; Li et al., 2023) |
| *Fhb-7EL* | | FHB | *Thinopyrum* | *Th. elongatum* | 2 | Medium | Moderate | Pre-breeding | Relationship to *Fhb7* unclear | (Guo et al., 2023b) |
| *Bdv2* | | BYDV | *Thinopyrum* | *Th. intermedium* | 2 | Medium | Moderate | Commercial (regional) | Large segment; limited multi-environmental yield data | (Larkin et al., 2002; Gao et al., 2009; Qonaah et al., 2026) |
| *Pm55* | | Powdery mildew | *Dasypyrum* | *D. villosum* | 2 | Medium | Moderate | Pre-breeding | Stage-specific resistance | (Lu et al., 2024) |
| *Yr4EL* | | Yellow rust | *Thinopyrum* | *Th. elongatum* | 2 | Medium | Moderate | Pre-breeding | Recent mapping; limited field data | (Gong et al., 2024, 2026) |
| *YrT14* | | Yellow rust | *Thinopyrum* | *Th. intermedium* | 2 | Medium | Moderate | Pre-breeding | Fine mapping incomplete | (Guo et al., 2023a) |
| 6Ag^i^2 | | Disease complex | *Thinopyrum* | *Th. intermedium* | 2 | Medium | Moderate | Regional (Russia) | Limited multi-env. yield data | (Salina et al., 2015; Ivanova et al., 2021) |
| T7DL•7E(Ag) | | Drought tolerance | *Thinopyrum* | *Th. elongatum* | 2 | Medium | Moderate | Pre-breeding | Root trait; field yield data pending | (Placido et al., 2013) |
| T4StS·1JvsS | | Drought tolerance | *Thinopyrum* | *Th. ponticum/intermedium* | 2 | High | Moderate | Pre-breeding | Single study with extensive phenotyping | (Kruppa et al., 2025) |
| *Septoria resistance* | | Septoria blotch | *Thinopyrum* | *Th. ponticum* | 2 | Medium | Moderate | Regional (Siberia) | Genes not identified | (Plotnikova et al., 2023b) |
| Tier 3 (53 introgressions, 75.7%) | | | | | | | | | | |
| 5Eb introgression | | Salinity tolerance | *Thinopyrum* | *Th. bessarabicum* | 3 | Low | Weak | Pre-breeding | Controlled conditions only | (King et al., 1996, 1997) |
| 3E(3D) substitution | | Salinity tolerance | *Thinopyrum* | *Th. elongatum* | 3 | Low | Weak | Pre-breeding | Seedling stage; no field yield | (Zeng et al., 2023) |
| *TtMYB1* | | Salinity tolerance | *Thinopyrum* | *Th. elongatum* | 3 | Low | Weak | Molecular candidate | In vitro/heterologous only | (Mu et al., 2024) |
| *TtLEA2-1* | | Salinity tolerance | *Thinopyrum* | *Th. elongatum* | 3 | Low | Weak | Pre-breeding | No field validation | (Yang et al., 2021; Peng et al., 2022) |
| *NHX/HKT* candidates | | Salinity tolerance | *Thinopyrum* | *Th. elongatum* via *Tritipyrum* | 3 | Low | Weak | Molecular candidate | Gene family studies; no introgression | (Li et al., 2022b, 2025c; Liu et al., 2024) |
| *D. villosum* addition lines | | Salinity tolerance | *Dasypyrum* | *D. villosum* | 3 | Low | Weak | Experimental | No field validation | (Zhong and Dvořák, 1995; Han et al., 2023) |
| 3St substitution | | Drought tolerance | *Thinopyrum* | *Th. ponticum* | 3 | Low | Weak | Experimental | Physiological data; no yield | (Türkösi et al., 2025) |
| *Hl1th* (pubescence) | | Drought tolerance | *Thinopyrum* | *Th. ponticum* | 3 | Low | Weak | Pre-breeding | Single report; no yield | (Simonov et al., 2024) |
| *4E addition* | | Perenniality | *Thinopyrum* | *Th. elongatum* | 3 | Low | Weak | Experimental | Cytogenetic stock | (Lammer, 2004) |
| *Sr68* | | Stem rust | *Thinopyrum* | *Th. junceum* | 3 | Low | Weak | Pre-breeding | Single report; no multi-env. | (Mandal et al., 2025) |
| *Sr52* | | Stem rust | *Dasypyrum* | *D. villosum* | 3 | Low | Weak | Pre-breeding | Temperature-sensitive; no field yield | (Li et al., 2019) |
| *Yr69* | | Yellow rust | *Thinopyrum* | *Th. ponticum* | 3 | Low | Weak | None | Not cloned; no multi-env. valid. | (Hou et al., 2016) |
| *YrTp1 / YrTp2* | | Yellow rust | *Thinopyrum* | *Th. ponticum* | 3 | Low | Weak | None | Single report; no independent valid. | (Yin et al., 2006) |
| *YrCH*-1BS translocation | | Yellow rust | *Thinopyrum* | *Th. intermedium* | 3 | Low | Weak | Pre-breeding | Single report; no independent valid. | (Zheng et al., 2020) |
| *YrCD-3* | | Yellow rust | *Dasypyrum* | *D. villosum* | 3 | Low | Weak | Pre-breeding | Single report; limited field screen | (Zhang et al., 2022a) |
| *Yr7VS* | | Yellow rust | *Dasypyrum* | *D. villosum* | 3 | Low | Weak | Pre-breeding | No independent validation | (Hou et al., 2024b) |
| *PmSN0293* | | Powdery mildew | *Thinopyrum* | *Th. ponticum* | 3 | Low | Weak | Pre-breeding | Gene not cloned; single report | (He et al., 2017; Li et al., 2022c) |
| *Pm62* | | Powdery mildew | *Dasypyrum* | *D. villosum* | 3 | Low | Weak | Pre-breeding | No independent validation | (Zhang et al., 2018) |
| *PmRc1* | | Powdery mildew | *Roegneria* | *R. ciliaris* | 3 | Low | Weak | Pre-breeding | Single report; no multi-env. | (Cheng et al., 2024) |
| *Fhb6* | | FHB | *Elymus* | *E. tsukushiensis* | 3 | Low | Weak | Pre-breeding | Greenhouse only; no yield | (Cainong et al., 2015) |
| *FhbRc1* | | FHB | *Roegneria* | *R. ciliaris* | 3 | Low | Weak | Pre-breeding | Single report | (Song et al., 2023) |
| *FhbRc2* | | FHB | *Roegneria* | *R. ciliaris* | 3 | Low | Weak | Pre-breeding | Single report | (Song et al., 2024) |
| Ns substitutions | | Stripe rust + FHB | *Leymus* | *L. mollis* | 3 | Low | Weak | Pre-breeding | Single population; no yield | (Zhao et al., 2019; Du et al., 2022) |
| *E. repens* translocation | | FHB + Yellow rust | *Elymus* | *E. repens* | 3 | Low | Weak | Pre-breeding | Single population; no yield | (Gong et al., 2019) |
| *Lr55* | | Leaf rust | *Elymus* | *E. trachycaulus* | 3 | Low | Weak | Pre-breeding | Not cloned; large segment; no yield | (Friebe et al., 2005; Wang et al., 2025) |
| *E. sibiricus* addition *(3St)* | | Leaf rust | *Elymus* | *E. sibiricus* | 3 | Low | Weak | Pre-breeding | Single report; adult plant resist. | (Motsnyi et al., 2024) |
| *CreV* | | Cyst nematode | *Dasypyrum* | *D. villosum* | 3 | Low | Weak | Pre-breeding | Greenhouse only | (Zhang et al., 2016) |
| Eyespot resistance (2V) | | Eyespot | *Dasypyrum* | *D. villosum* | 3 | Low | Weak | Pre-breeding | Limited field screen | (Liu et al., 2023) |
| 1Ns(1D) substitution | | Grain quality | *Leymus* | *L. mollis* | 3 | Low | Weak | Pre-breeding | Single environment evaluation | (Li et al., 2025a) |
| *1Ey HMW-GS* | | Grain quality | *Thinopyrum* | *Th. elongatum* | 3 | Low | Weak | Pre-breeding | Single environment; no multi-env. yield | (Dai et al., 2023) |
| *PsyE1* (carotenoids) | | Grain quality | *Thinopyrum* | *Th. ponticum* | 3 | Low | Weak | Regional/Historical | Yield penalty in some environments | (Kato et al., 2025) |
| *Ba1* (anthocyanin) | | Grain quality | *Thinopyrum* | *Th. ponticum* | 3 | Low | Weak | Experimental | Pleiotropic effects on yield | (Gordeeva et al., 2022) |
| 6VbS chromatin | | Yield components | *Dasypyrum* | *D. breviaristatum* | 3 | Low | Weak | Experimental | Cytogenetic line only; no yield data | (Jiang et al., 2022) |
| 1ScS•1AL translocation | | Yield components | *Roegneria* | *R. ciliaris* | 3 | Low | Weak | Pre-breeding | Single study; limited replication | (Cheng et al., 2024) |
| ×*Trititrigia* (grain quality) | | Grain quality | *Thinopyrum* | *Th. intermedium* and/or *Th. ponticum* | 3 | Low | Weak | Regional (Russia) | Lower yield potential vs. wheat; anthocyanin biofortification potential in some lines | (Lachuga et al., 2023; Shchuklina et al., 2026) |
| 4Jr/St substitution | | Grain quality/yield | *Thinopyrum* | *Th. intermedium* | 3 | Low | Weak | Pre-breeding | Single-location yield data | (Wu et al., 2025) |
| 7E segmental introgression | Salinity tolerance | | *Thinopyrum* | *Th. elongatum / Th. ponticum* | 3 | Low | Weak | Pre-breeding | Single study; durum wheat background | (Tounsi et al., 2024) |
| 3St substitution (salinity) | Salinity tolerance | | *Thinopyrum* | *Th. intermedium × Th. ponticum* | 3 | Low | Weak | Experimental | Seedling phenotyping; no field yield | (Gholizadeh et al., 2026) |
| 5V introgression (drought) | Drought tolerance | | *Dasypyrum* | *D. villosum* | 3 | Low | Weak | Experimental | Greenhouse/lysimeter; complex translocation | (Djanaguiraman et al., 2019) |
| *Pm3VS* | Powdery mildew | | *Dasypyrum* | *D. villosum* | 3 | Low | Weak | Pre-breeding | Single study; no independent validation | (Hou et al., 2024a) |
| *Pm4VL* | Powdery mildew | | *Dasypyrum* | *D. villosum* | 3 | Low | Weak | Pre-breeding | Single study | (Wei et al., 2024) |
| *Pm67* | Powdery mildew | | *Dasypyrum* | *D. villosum* | 3 | Low | Weak | Pre-breeding | No independent validation | (Zhang et al., 2021) |
| *Pm5V / Yr5V* | Powdery mildew + yellow rust | | *Dasypyrum* | *D. villosum* | 3 | Low | Weak | Pre-breeding | Single study; fine-mapped | (Zhang et al., 2022b) |
| *Lr* (7Js) | Leaf rust | | *Thinopyrum* | *Th. intermedium* | 3 | Low | Weak | Pre-breeding | Single study; no multi-env. | (Yang et al., 2023) |
| DS5Ag(5D) substitution | Powdery mildew + leaf rust | | *Thinopyrum* | *Th. ponticum* | 3 | Low | Weak | Pre-breeding | Single study; one environment | (Zhang et al., 2024) |
| T3AL·7StS-7StL translocation | Yellow rust | | *Thinopyrum* | *Th. intermedium* | 3 | Low | Weak | Pre-breeding | Single report; linked to Lr | (Li et al., 2025b) |
| *Yr* (4JSL) | Yellow rust | | *Thinopyrum* | *Th. intermedium* | 3 | Low | Weak | Pre-breeding | Chromosome arm locus; no gene cloned | (Li et al., 2022a) |
| *Yr* (2StS) | Yellow rust | | *Thinopyrum* | *Th. intermedium* | 3 | Low | Weak | Pre-breeding | Single study; no independent validation; no multi-environment field data | (Jiang et al., 2025) |
| 7St addition (*Pm*) | Powdery mildew | | *Thinopyrum* | *Th. ponticum* | 3 | Low | Weak | Experimental | Addition line; no field yield data; no agronomic validation | (Cheng et al., 2025) |
| 7Vb addition (*Yr*) | Yellow rust | | *Dasypyrum* | *D. breviaristatum* | 3 | Low | Weak | Experimental | Disomic addition line; no field data; purely experimental | (Li et al., 2016) |
| T3DS-3AS.3AL-7StS translocation | Leaf rust | | *Thinopyrum* | *Th. intermedium* | 3 | Low | Weak | Pre-breeding | Single report; no independent validation; no agronomic evaluation | (Li et al., 2025b) |
| Partial amphiploid 92048 | FHB + yellow rust | | *Thinopyrum* | *Th. intermedium* | 3 | Low | Weak | Experimental | Partial amphiploid; no yield evaluation; complex genomic constitution | (Luo et al., 2024) |
| *Lr29* | Leaf rust | | *Thinopyrum* | *Th. elongatum* | 3 | Low | Weak | Pre-breeding | Effective resistance source, but lacks documented large-scale commercial deployment and multi-environment agronomic validation; not widely used in modern cultivars despite historical availability | (Sears, 1973) |

**UNIFIED NOTES FOR SUPPLEMENTARY TABLES S1, S2, S3**

**1. FIELD VALIDATION**

High – ≥3 environments and ≥2 years with yield assessment or widely deployed in commercial cultivars with documented multi‑year efficacy

Medium – limited multi‑environment trials (≤2 environments or ≤2 years)

Low – controlled or single‑environment studies without yield data

Very low – in silico, in vitro, or molecular characterization only (no wheat introgression)

**2. EVIDENCE STRENGTH (formal definition)**

Strong – (i) cloned gene + High field validation + ≥2 independent studies, or (ii) locus with diagnostic markers, High field validation, commercial deployment, and confirmed by ≥2 independent studies

Moderate – any two of: (cloned/fine‑mapped), field validation Medium, independent validation

Weak – only one of the above, or preliminary data

Although correlated in practice, the two dimensions (field validation and evidence strength) are conceptually separated to avoid conflating experimental replication with genetic resolution.

**3. TRANSLATION LEVEL (for S1, S3)**

Breeding – deployed in commercial/regional cultivars with demonstrated agronomic impact

Pre‑breeding – introgressed into wheat but not yet commercially deployed or linkage drag constraints partially resolved

Pre‑commercial = advanced pre‑breeding stage with demonstrated field potential but not yet in commercial cultivars

Physiological – trait measured at physiological level without confirmed yield impact

Experimental – limited to experimental lines without agronomic validation

Molecular – in vitro/heterologous expression or in silico characterization only, not yet introgressed as validated trait

**4. DEPLOYMENT STATUS (for Main Table 1 and S3 uses «Translation level»)**

Commercial – registered cultivar(s) with documented area (e.g., *Pm21* >4M ha)

Regional – used in breeding or released in specific country/region

Pre‑breeding – introgressed into wheat but not yet in commercial cultivar

Pre‑commercial – advanced pre‑breeding with field potential

None – no known deployment

Deployment status reflects documented cultivar release, not adoption scale or agronomic importance.

**5. SEGMENT SIZE (for S2)**

Large – whole chromosome arm or >50 Mb (original introgression)

Medium – sub‑arm region or 10–50 Mb

Small – <10 Mb or cloned gene (often after engineering)

Unknown – not characterized

Note: Segment sizes are approximate and may vary among introgression lines depending on recombination history, cytogenetic background, and donor species. «Engineered» refers to reduced segments obtained via induced homoeologous recombination or chromosome engineering.

FL (fraction length): relative position on chromosome arm (0 = centromere, 1 = telomere).

**6. MARKER TYPE (for S2)**

Diagnostic – co‑segregating or gene‑specific markers suitable for MAS (tightly linked, <1 cM)

Linked – markers at >1 cM distance, not fully diagnostic

Functional – derived from cloned gene sequence

**7. INCLUSION CRITERIA FOR MAIN TABLE 1**

Inclusion in Main Table requires at least Moderate evidence strength and field validation ≥Medium.

Exceptions may be included due to historical relevance or lack of alternative sources.

Loci with only Weak evidence or Low field validation are confined to Supplementary Table S2.

Tier 3 loci (*Yr69, YrTp1/YrTp2, CreV*) are included as illustrative examples of weak/contradictory evidence that populate the literature. Their presence highlights the gap between reported introgressions and validated breeding targets – a central theme of this review. They are not recommended as breeding targets. These loci are included for completeness of reported literature space, not prioritization.

**8. TEMPERATURE SENSITIVITY**

For *Sr52* and *Wsm1*, efficacy drops at temperatures above ~20°C (Wsm1 ineffective above ~20°C; *Sr52* partially effective at 24°C, ineffective at 28°C), limiting field performance in warm climates.

**9. LARGE SEGMENTS AND COMMERCIAL SUCCESS**

Although small segment size and minimal linkage drag generally favour introgression success, large whole‑arm introgressions (e.g., *Pm21, Bdv2, Wsm1*) can be successful when the target gene has a major effect and/or shortened recombinants are available (e.g., *Wsm1* rec213).

**10. E‑GENOME NOMENCLATURE (FOR S2)**

E‑genome chromosomes are denoted as 7E, 7el, 7Ae, or 7Ag depending on species and historical nomenclature; these refer to homoeologous chromosomes of *Thinopyrum* spp.

**11. SPECIFIC NOTES FOR SELECTED LOCI**

*Fhb7*: A novel allele (Fhb7^The2^) without yellow pigment linkage drag was reported by Zhang et al. (2022c), but this is not yet widely deployed. The original Fhb7^Thp^ allele retains linkage drag. The tight linkage between *Fhb7* and *PSY-E2* was broken via homoeologous recombination in specific lines (Li et al., 2023). Background‑dependent effects have been noted, but no widely accepted peer‑reviewed evidence contradicts the gene's overall functionality.

*Lr19*: Kuzmanović et al. (2018) conducted multi‑location field trials (9 trials across contrasting environments) validating the agronomic potential of engineered lines. Xu et al. (2023) reported breaking linkage with yellow pigment in one translocation line, but the gene is not yet widely used in commercial cultivars.

*Wsm1*: Field efficacy is confirmed in multi‑environment trials. The original translocation carries a yield penalty in the absence of the virus (Sharp et al., 2002); shorter recombinants (e.g., rec213) have been developed to mitigate this issue (Guttieri et al., 2023). Commercial cultivars Mace and RonL carry *Wsm1* but resistance is temperature‑sensitive (>20°C).

*Fhb7* vs *Fhb-7EL* (in Table S1): *Fhb7* originates from *Th. ponticum* (7el2L, GST gene, HGT origin); *Fhb-7EL* from *Th. elongatum* (7EL). Current evidence does not confirm homology; they may represent distinct loci or alleles. Listed separately to avoid overclaiming. Functional equivalence is not assumed despite potential convergent resistance phenotypes.

*Sr43*: Original introgression from *Th. ponticum* (2n=10x=70) with large segment; gene cloned from *Th. elongatum* (2n=2x=14). Engineered lines have reduced segment (~13–17% of arm) but linkage drag not fully eliminated.

**12. NOTES SPECIFIC TO SUPPLEMENTARY TABLE S3**

Purpose: This table maps reported effects of wild Triticeae introgressions on grain quality, nutritional traits, and yield trade‑offs. It is designed as a pre‑breeding reference rather than a strict causal statement. Effects are often background‑dependent, and trade‑offs may vary with genetic background, segment length, and environment.

Important caveats:

– For HMW‑GS introgressions (e.g., 1Ey from *Th. elongatum*), effects on yield and quality are background‑dependent (influenced by substitution type, Glu‑D1 alleles, and segment structure).

– Yield penalties reported for *Lr19*‑associated *PsyE1* are not universal; shortened recombinants can reduce linkage drag.

– Single‑study estimates (e.g., *Roegneria* *ciliaris* +15–20% spikelets) should be treated as provisional until validated across environments.

– ×*Trititrigia* yield comparisons involve different growing conditions and management; direct yield penalty attribution is confounded.

This table is a map of effects, not a definitive causality statement. It is intended to support hypothesis generation and pre‑breeding decisions, not to replace multi‑environment validation.

**13. NOTES SPECIFIC TO SUPPLEMENTARY TABLE S4**

S4 is a complete inventory of reviewed wild Triticeae introgressions. It includes alien gene transfers, chromosome addition/substitution/translocation lines, and partial amphiploids proposed as trait donors. Synthetic amphiploids (×*Trititrigia*, *Tritipyrum*) are classified under Thinopyrum, with their donor species field indicating the *Thinopyrum* genome source and the amphiploid form. The “Key limitation” column indicates the primary translational barrier preventing a higher Tier. All tier definitions and evidence criteria follow the Methods section and Unified Notes above. The total number of inventoried introgressions (n=70) reflects the comprehensive scope of the review, while the percentages in tier headings reflect the distribution of evidence levels across this full set.

**14. SCOPE AND EXCEPTIONS**

*Aegilops* species are not included in these tables (see review scope). Although *Aegilops* species are generally excluded from this review (due to higher genomic compatibility), the specific line «Drought tolerance» in Supplementary Table S1 is included as an exceptional case because it combines a tertiary gene pool donor (*D. villosum*) with an *Aegilops* segment in a composite translocation (Djanaguiraman et al., 2019). Loci present in Supplementary Table S2 but absent from Main Table 1 (e.g., *Yr4EL*) are either too recent or lack sufficient multi‑environment validation for inclusion in the main synthesis; they are provided here as additional context. Supplementary Table S4 provides the complete inventory of all reviewed introgressions, whereas Main Table 1 is restricted to those with at least Moderate evidence strength and field validation ≥Medium (see Methods and Note 7).

**15. ONTOLOGY HIERARCHY FOR GENETIC UNITS**

Entity hierarchy rule (for classification in tables):

- Gene – cloned, functional validation (protein/DNA level)

- Locus – mapped region, gene unknown, but consistent genetic evidence

- QTL – statistical association, often broad interval, candidate genes not confirmed

- Segment – defined alien chromatin (cytogenetic), no single gene identified

These are categorical evidence entities, not directly comparable metrics. Their hierarchy reflects increasing genetic resolution, not necessarily breeding value. All categories are reported within a single framework for traceability of reported literature claims, not for direct quantitative comparison across categories.

**16. EFFECT SIZE CAVEAT (for all tables)**

Quantitative values (e.g., yield effects, protein content, thousand‑kernel weight) are reported as published in the original studies. They are not universal constants and may not transfer directly to all breeding backgrounds due to genotype‑by‑environment interactions, differences in experimental design, and genetic background. Effect sizes should be interpreted as qualitative indicators of direction and approximate magnitude, not as precise predictions.

**17. PUBLICATION BIAS**

The reported effect sizes are as published in the original studies. As in many fields of plant science, positive results may be overrepresented due to publication bias (i.e., studies reporting successful introgressions or positive effects are more likely to be published than those reporting neutral or negative outcomes). Grey literature from breeding companies was not accessible. Therefore, the distribution of evidence in these tables should be interpreted as a qualitative synthesis, not as a precise estimate of true success rates.

**References**

Cainong, J. C., Bockus, W. W., Feng, Y., Chen, P., Qi, L., Sehgal, S. K., et al. (2015). Chromosome engineering, mapping, and transferring of resistance to Fusarium head blight disease from *Elymus tsukushiensis* into wheat. *Theor. Appl. Genet.* 128, 1019–1027. doi: 10.1007/s00122-015-2485-1

Cheng, M., Zhang, H., Zhang, Y., Tang, X., Wang, Z., Zhang, X., et al. (2024). Cytological mapping of a powdery mildew resistance locus *PmRc1* based on wheat-*Roegneria ciliaris* structural rearrangement library. *Theor. Appl. Genet.* 137, 276. doi: 10.1007/s00122-024-04768-w

Cheng, X., Guan, Y., Zhao, J., Yang, X., Wang, G., Li, T., et al. (2025). Establishment of a set of St-group wheat-*Thinopyrum ponticum* derivative lines conferring resistance to powdery mildew. *Front. Plant Sci.* 16, 1576050. doi: 10.3389/fpls.2025.1576050

Dai, Y., Li, J., Shi, J., Gao, Y., Ma, H., Wang, Y., et al. (2023). Molecular Characterization and Marker Development of the *HMW-GS* Gene from *Thinopyrum elongatum* for Improving Wheat Quality. *Int. J. Mol. Sci.* 24, 11072. doi: 10.3390/ijms241311072

Djanaguiraman, M., Prasad, P. V. V., Kumari, J., Sehgal, S. K., Friebe, B., Djalovic, I., et al. (2019). Alien chromosome segment from *Aegilops speltoides* and *Dasypyrum villosum* increases drought tolerance in wheat via profuse and deep root system. *BMC Plant Biol.* 19, 242. doi: 10.1186/s12870-019-1833-8

Du, X., Feng, X., Li, R., Jin, Y., Shang, L., Zhao, J., et al. (2022). Cytogenetic identification and molecular marker development of a novel wheat-*Leymus mollis* 4Ns(4D) alien disomic substitution line with resistance to stripe rust and Fusarium head blight. *Front. Plant Sci.* 13, 1012939. doi: 10.3389/fpls.2022.1012939

Du, X., Jin, Y., Xu, X., Wang, Y., Li, T., Zhao, J., et al. (2026). Comparative molecular cytogenetic analysis and molecular marker development of wheat–*Leymus mollis* and wheat-*Psathyrostachys huashanica* 7Ns alien chromosome lines. *J. Integr. Agric.*, S2095311926000298. doi: 10.1016/j.jia.2026.01.028

Dundas, I. S., Anugrahwati, D. R., Verlin, D. C., Park, R. F., Bariana, H. S., Mago, R., et al. (2007). New sources of rust resistance from alien species: meliorating linked defects and discovery. *Aust. J. Agric. Res.* 58, 545–549. doi: 10.1071/AR07056

Dundas, I., Zhang, P., Verlin, D., Graner, A., and Shepherd, K. (2015). Chromosome Engineering and Physical Mapping of the *Thinopyrum ponticum* Translocation in Wheat Carrying the Rust Resistance Gene *Sr26*. *Crop Sci.* 55, 648–657. doi: 10.2135/cropsci2014.08.0590

Feng, X., Du, X., Wang, S., Deng, P., Wang, Y., Shang, L., et al. (2022). Identification and DNA Marker Development for a Wheat-*Leymus mollis* 2Ns (2D) Disomic Chromosome Substitution. *Int. J. Mol. Sci.* 23, 2676. doi: 10.3390/ijms23052676

Friebe, B., Wilson, D., Raupp, W., Gill, B., and Brown-Guedira, G. (2005). Notice of release of KS04WGRC45 leaf rust-resistant hard white winter wheat germplasm. *Annu. Wheat Newsl.* 51, 188–189.

Gao, L., Ma, Q., Liu, Y., Xin, Z., and Zhang, Z. (2009). Molecular characterization of the genomic region harboring the BYDV-resistance gene *Bdv2* in wheat. *J. Appl. Genet.* 50, 89–98. doi: 10.1007/BF03195659

Gholizadeh, F., Janda, T., Varga, B., György, M., Molnár, I., Kruppa, K., et al. (2026). Genotype-dependent salt tolerance mechanisms in wheat–*Thinopyrum* introgression lines revealed by ion transporter gene expression and seedling phenotyping. *Sci. Rep.* 16, 7647. doi: 10.1038/s41598-026-40421-w

Gong, B., Gao, J., Xie, Y., Zhang, H., Zhu, W., Xu, L., et al. (2024). Development of wheat-tetraploid *Thinopyrum elongatum* 4EL small fragment translocation lines with stripe rust resistance gene *Yr4EL*. *Theor. Appl. Genet.* 137, 246. doi: 10.1007/s00122-024-04756-0

Gong, B., Zhang, H., Lu, Y., Chen, L., Zhu, W., Xu, L., et al. (2026). Fine Mapping of the All‐Stage Stripe Rust Resistance Gene *Yr4EL* and Its Utilization in Wheat Resistance Breeding. *Plant Cell Environ.* 49, 1064–1077. doi: 10.1111/pce.70278

Gong, B., Zhu, W., Li, S., Wang, Y., Xu, L., Wang, Y., et al. (2019). Molecular cytogenetic characterization of wheat–*Elymus repens* chromosomal translocation lines with resistance to Fusarium head blight and stripe rust. *BMC Plant Biol.* 19, 590. doi: 10.1186/s12870-019-2208-x

Gordeeva, E., Shoeva, O., Mursalimov, S., Adonina, I., and Khlestkina, E. (2022). Fine Points of Marker-Assisted Pyramiding of Anthocyanin Biosynthesis Regulatory Genes for the Creation of Black-Grained Bread Wheat (*Triticum aestivum* L.) Lines. *Agronomy* 12, 2934. doi: 10.3390/agronomy12122934

GrainGenes (n. d.). MASWheat: Rusts resistance genes *Lr19*, *Sr25* and color gene *Y*. Available online at: https://graingenes.org/GG3/content/maswheat-rusts-resistancegenes-lr19-sr25-and-color-gene-y.

Guo, J., Zhang, X., Hou, Y., Cai, J., Shen, X., Zhou, T., et al. (2015). High-density mapping of the major FHB resistance gene *Fhb7* derived from *Thinopyrum ponticum* and its pyramiding with *Fhb1* by marker-assisted selection. *Theor. Appl. Genet.* 128, 2301–2316. doi: 10.1007/s00122-015-2586-x

Guo, X., Huang, Y., Wang, J., Fu, S., Wang, C., Wang, M., et al. (2023a). Development and cytological characterization of wheat–*Thinopyrum intermedium* translocation lines with novel stripe rust resistance gene. *Front. Plant Sci.* 14, 1135321. doi: 10.3389/fpls.2023.1135321

Guo, X., Shi, Q., Liu, Y., Su, H., Zhang, J., Wang, M., et al. (2023b). Systemic development of wheat–*Thinopyrum elongatum* translocation lines and their deployment in wheat breeding for Fusarium head blight resistance. *Plant J.* 114, 1475–1489. doi: 10.1111/tpj.16190

Guo, X., Shi, Q., Wang, M., Yuan, J., Zhang, J., Wang, J., et al. (2023c). Functional analysis of the glutathione S‐transferases from *Thinopyrum* and its derivatives on wheat Fusarium head blight resistance. *Plant Biotechnol. J.* 21, 1091–1093. doi: 10.1111/pbi.14021

Guttieri, M. J., Bowden, R. L., Zhang, G., Haley, S., Frels, K., Hein, G. L., et al. (2023). Agronomic and quality impact of a shortened translocation for *wheat streak mosaic virus* resistance. *Crop Sci.* 63, 622–634. doi: 10.1002/csc2.20876

Han, Z., Jia, Z., Liang, Q., Wang, K., Tang, H., Ye, X., et al. (2023). Salt Tolerance at Seedling Stage and Analysis of Selenium and Folic Acid Content in Seeds in Two Sets of Wheat-*Dasypyrum villosum* Chromosome Additional Lines. *Biotechnol. Bull.* 39, 185–193. doi: 10.13560/j.cnki.biotech.bull.1985.2023-0003

He, F., Bao, Y., Qi, X., Ma, Y., Li, X., and Wang, H. (2017). Molecular cytogenetic identification of a wheat–*Thinopyrum ponticum* translocation line resistant to powdery mildew. *J. Genet.* 96, 165–169. doi: 10.1007/s12041-017-0754-2

Hohmann, U., Busch, W., Badaeva, K., Friebe, B., and Gill, B. S. (1996). Molecular cytogenetic analysis of *Agropyron* chromatin specifying resistance to barley yellow dwarf virus in wheat. *Genome* 39, 336–347. doi: 10.1139/g96-044

Hou, F., Chen, H., Zhang, T., Jin, Y., Kong, L., Liu, X., et al. (2024a). Introgression of an All-Stage and Broad-Spectrum Powdery Mildew Resistance Gene *Pm3VS* from *Dasypyrum villosum* Chromosome 3V into Wheat. *Plant Dis.* 108, 2073–2080. doi: 10.1094/PDIS-11-23-2495-RE

Hou, F., Jin, Y., Hu, J., Kong, L., Liu, X., Xing, L., et al. (2024b). Transferring an Adult-Plant Stripe-Rust Resistance Gene *Yr7VS* from Chromosome 7V of *Dasypyrum villosum* (L.) to Bread Wheat. *Plants* 13, 1875. doi: 10.3390/plants13131875

Hou, L., Jia, J., Zhang, X., Li, X., Yang, Z., Ma, J., et al. (2016). Molecular Mapping of the Stripe Rust Resistance Gene *Yr69* on Wheat Chromosome 2AS. *Plant Dis.* 100, 1717–1724. doi: 10.1094/PDIS-05-15-0555-RE

Ivanova, Y. N., Rosenfread, K. K., Stasyuk, A. I., Skolotneva, E. S., and Silkova, O. G. (2021). Raise and characterization of a bread wheat hybrid line (Tulaykovskaya 10 × Saratovskaya 29) with chromosome 6Ag^i^2 introgressed from *Thinopyrum intermedium*. *Vavilovskii Zhurnal Genetiki i Selektsii* 25, 701–712. doi: 10.18699/VJ21.080

Jiang, C., Jiang, W., Liu, M., Wang, H., Yang, E., Yang, Z., et al. (2022). Characterization of a Wheat-*Dasypyrum breviaristatum* Chromosome Addition and Its Derived Progenies Carrying Novel *Dasypyrum*-Specific Gliadin Genes. *Agronomy* 12, 1673. doi: 10.3390/agronomy12071673

Jiang, C., Luo, Y., Huang, D., Chen, M., Yang, E., Li, G., et al. (2025). Characterization of a New Stripe Rust Resistance Gene on Chromosome 2StS from *Thinopyrum intermedium* in Wheat. *Plants* 14, 1538. doi: 10.3390/plants14101538

Kato, K., Ban, Y., Yanaka, M., Yoshioka, M., Okusu, H., Tanaka, T., et al. (2025). *Psy-E1* derived from *Thinopyrum ponticum* contributes strong yellowness to durum wheat but may cause yield loss in Japan. *Breed. Sci.* 75, 93–101. doi: 10.1270/jsbbs.24070

Khokhar, J. S., Sareen, S., Tyagi, B. S., Wilson, L., Young, S., King, J., et al. (2020). Novel Sources of Variation in Grain Yield, Components and Mineral Traits Identified in Wheat Amphidiploids Derived from *Thinopyrum bessarabicum* (Savul. & Rayss) Á. Löve (Poaceae) under Saline Soils in India. *Sustainability* 12, 8975. doi: 10.3390/su12218975

King, I. P., Forster, B. P., Law, C. C., Cant, K. A., Orford, S. E., Gorham, J., et al. (1997). Introgression of salt‐tolerance genes from *Thinopyrum bessarabicum* into wheat. *New Phytol.* 137, 75–81. doi: 10.1046/j.1469-8137.1997.00828.x

King, I. P., Orford, S. E., Cant, K. A., Reader, S. M., and Miller, T. E. (1996). An assessment of the salt tolerance of wheat/*Thinopyrum bessarabicum* 5E^b^ addition and substitution lines. *Plant Breed.* 115, 77–78. doi: 10.1111/j.1439-0523.1996.tb00876.x

Knott, D. R. (1961). The inheritance of rust resistance. vi. the transfer of stem rust resistance from *Agropyron elongatum* to common wheat. *Can. J. Plant Sci.* 41, 109–123. doi: 10.4141/cjps61-014

Knott, D. R. (1968). Translocations involving *Triticum* chromosomes and *Agropyron* chromosomes carrying rust resistance. *Can. J. Genet. Cytol.* 10, 695–696. doi: 10.1139/g68-087

Kruppa, K., Türkösi, E., Holušová, K., Kalapos, B., Szakács, É., Cséplő, M., et al. (2025). Genotyping-by-sequencing uncovers a *Thinopyrum* 4StS·1JvsS Robertsonian translocation linked to multiple stress tolerances in bread wheat. *Theor. Appl. Genet.* 138, 13. doi: 10.1007/s00122-024-04791-x

Kuzmanović, L., Ruggeri, R., Able, J. A., Bassi, F. M., Maccaferri, M., Tuberosa, R., et al. (2018). Yield of chromosomally engineered durum wheat-*Thinopyrum ponticum* recombinant lines in a range of contrasting rain-fed environments. *Field Crops Res.* 228, 147–157. doi: 10.1016/j.fcr.2018.08.014

Lachuga, Y., Meskhi, B., Pakhomov, V., Semenikhina, Y., Kambulov, S., Rudoy, D., et al. (2023). Experience in the Cultivation of a New Perennial Cereal Crop—*Trititrigia* in the Conditions of South of the Rostov Region. *Agriculture* 13, 605. doi: 10.3390/agriculture13030605

Lammer, D. (2004). A single chromosome addition from *Thinopyrum elongatum* confers a polycarpic, perennial habit to annual wheat. *J. Exp. Bot.* 55, 1715–1720. doi: 10.1093/jxb/erh209

Larkin, P., Kleven, S., and Banks, P. (2002). Utilizing *Bdv2*, the *Thinopyrum intermedium* source of BYDV resistance, to develop wheat cultivars. *Recent Advances and Future Strategies*, 60–63. Mexico: CIMMYT.

Li, G., Chen, Q., Jiang, W., Zhang, A., Yang, E., and Yang, Z. (2022a). Molecular and Cytogenetic Identification of Wheat-*Thinopyrum intermedium* Double Substitution Line-Derived Progenies for Stripe Rust Resistance. *Plants* 12, 28. doi: 10.3390/plants12010028

Li, G., Gao, D., Zhang, H., Li, J., Wang, H., La, S., et al. (2016). Molecular cytogenetic characterization of *Dasypyrum breviaristatum* chromosomes in wheat background revealing the genomic divergence between *Dasypyrum* species. *Mol. Cytogenet.* 9, 6. doi: 10.1186/s13039-016-0217-0

Li, H., Dong, Z., Ma, C., Tian, X., Qi, Z., Wu, N., et al. (2019). Physical mapping of stem rust resistance gene *Sr52* from *Dasypyrum villosum* based on *ph1b*-induced homoeologous recombination. *Int. J. Mol. Sci.* 20, 4887. doi: 10.3390/ijms20194887

Li, J., Guan, H., Wang, Y., Dong, C., Trethowan, R., McIntosh, R. A., et al. (2024). Cytological and molecular characterization of wheat lines carrying leaf rust and stem rust resistance genes *Lr24* and *Sr24*. *Sci. Rep.* 14, 12816. doi: 10.1038/s41598-024-63835-w

Li, J., Li, J., Cheng, X., Zhao, L., Yang, Z., Wu, J., et al. (2021). Molecular Cytogenetic and Agronomic Characterization of the Similarities and Differences Between Wheat–*Leymus mollis* Trin. and Wheat–*Psathyrostachys huashanica* Keng 3Ns (3D) Substitution Lines. *Front. Plant Sci.* 12, 644896. doi: 10.3389/fpls.2021.644896

Li, J., Liang, B., Huo, W., Yuan, J., Tang, X., Yang, H., et al. (2025a). Introducing chromosome 1Ns from *Leymus mollis* improves grain protein content of recipient wheat. *Plant Cell Rep.* 44, 255. doi: 10.1007/s00299-025-03641-4

Li, J., Ryan, M., Dong, C., Forrest, K. L., Hayden, M. J., Singh, S., et al. (2025b). Pseudo-linkage or real-linkage of rust resistance genes in a wheat-*Thinopyrum intermedium* translocation line. *Theor. Appl. Genet.* 138, 15. doi: 10.1007/s00122-024-04807-6

Li, K., Cong, C., Wang, Y., Zhang, H., Li, Y., Xiao, J., et al. (2025c). Genome-wide analysis of the *Tritipyrum* bHLH gene family and the response of *TtbHLH310* in salt-tolerance. *BMC Genomics* 26, 549. doi: 10.1186/s12864-025-11657-z

Li, K., Liu, X., He, F., Chen, S., Zhou, G., Wang, Y., et al. (2022b). Genome-wide analysis of the *Tritipyrum* WRKY gene family and the response of *TtWRKY256* in salt-tolerance. *Front. Plant Sci.* 13, 1042078. doi: 10.3389/fpls.2022.1042078

Li, M., Yuan, Y., Ni, F., Li, X., Wang, H., and Bao, Y. (2022c). Characterization of Two Wheat-*Thinopyrum ponticum* Introgression Lines With Pyramiding Resistance to Powdery Mildew. *Front. Plant Sci.* 13, 943669. doi: 10.3389/fpls.2022.943669

Li, X., Li, D., Xuan, Y., He, Z., Zhao, L., Hao, Y., et al. (2023). Elimination of the yellow pigment gene *PSY-E2* tightly linked to the Fusarium head blight resistance gene *Fhb7* from *Thinopyrum ponticum*. *Crop J.* 11, 957–962. doi: 10.1016/j.cj.2022.12.005

Liu, C., Guo, W., Wang, Y., Fu, B., Doležel, J., Liu, Y., et al. (2023). Introgression of sharp eyespot resistance from *Dasypyrum villosum* chromosome 2VL into bread wheat. *Crop J.* 11, 1512–1520. doi: 10.1016/j.cj.2023.04.013

Liu, S., Yu, L.-X., Singh, R. P., Jin, Y., Sorrells, M. E., and Anderson, J. A. (2010). Diagnostic and co-dominant PCR markers for wheat stem rust resistance genes *Sr25* and *Sr26*. *Theor. Appl. Genet.* 120, 691–697. doi: 10.1007/s00122-009-1186-z

Liu, X., Zhou, G., Chen, S., Jia, Z., Zhang, S., He, F., et al. (2024). Genome-wide analysis of the *Tritipyrum* NAC gene family and the response of *TtNAC477* in salt tolerance. *BMC Plant Biol.* 24, 40. doi: 10.1186/s12870-023-04629-6

Lu, C., Du, J., Chen, H., Gong, S., Jin, Y., Meng, X., et al. (2024). Wheat *Pm55* alleles exhibit distinct interactions with an inhibitor to cause different powdery mildew resistance. *Nat. Commun.* 15, 503. doi: 10.1038/s41467-024-44796-0

Luo, X., He, Y., Feng, X., Huang, M., Huang, K., Li, X., et al. (2024). Molecular and Cytological Identification of Wheat-*Thinopyrum intermedium* Partial Amphiploid Line 92048 with Resistance to Stripe Rust and Fusarium Head Blight. *Plants* 13, 1198. doi: 10.3390/plants13091198

Mandal, S. N., Gill, B. K., Niu, Z., Zhang, Q., Klindworth, D. L., Bataller, S., et al. (2025). Introgression of stem rust resistance gene *Sr68* from *Thinopyrum junceum* into wheat. *Theor. Appl. Genet.* 138, 229. doi: 10.1007/s00122-025-05005-8

Motsnyi, I. I., Halaiev, O. V., Alіeksіeіeva, T. G., Chebotar, G. O., Chebotar, S. V., Betekhtin, A., et al. (2024). Cytogenetic and molecular identification of novel wheat-*Elymus sibiricus* addition lines with resistance to leaf rust and the presence of leaf pubescence trait. *Front. Plant Sci.* 15, 1482211. doi: 10.3389/fpls.2024.1482211

Mu, Y., Shi, L., Tian, H., Tian, H., Zhang, J., Zhao, F., et al. (2024). Characterization and transformation of *TtMYB1* transcription factor from *Tritipyrum* to improve salt tolerance in wheat. *BMC Genomics* 25, 163. doi: 10.1186/s12864-024-10051-5

Niu, Z., Klindworth, D. L., Yu, G., L Friesen, T., Chao, S., Jin, Y., et al. (2014). Development and characterization of wheat lines carrying stem rust resistance gene *Sr43* derived from *Thinopyrum ponticum*. *Theor. Appl. Genet.* 127, 969–980. doi: 10.1007/s00122-014-2272-4

Padhy, A. K., Kaur, P., Singh, B., Kaur, R., Bhatia, S., Shamshad, M., et al. (2023). In silico characterization of *Thinopyrum elongatum*-derived *PsyE1* gene and validation in 7D/7E bread wheat introgression lines open avenues for carotenoid biofortification in wheat. *Cereal Res. Commun.* 51, 75–85. doi: 10.1007/s42976-022-00279-w

Peng, Z., Wang, Y., Geng, G., Yang, R., Yang, Z., Yang, C., et al. (2022). Comparative Analysis of Physiological, Enzymatic, and Transcriptomic Responses Revealed Mechanisms of Salt Tolerance and Recovery in *Tritipyrum*. *Front. Plant Sci.* 12, 800081. doi: 10.3389/fpls.2021.800081

Pietrusińska, A., and Tyrka, M. (2021). Linkage of *Lr55* wheat leaf rust resistance gene with microsatellite and DArT-based markers. *Physiol. Mol. Plant Pathol.* 115, 101674. doi: 10.1016/j.pmpp.2021.101674

Placido, D. F., Campbell, M. T., Folsom, J. J., Cui, X., Kruger, G. R., Baenziger, P. S., et al. (2013). Introgression of Novel Traits from a Wild Wheat Relative Improves Drought Adaptation in Wheat. *Plant Physiol.* 161, 1806–1819. doi: 10.1104/pp.113.214262

Plotnikova, L., Knaub, V., and Pozherukova, V. (2023a). Nonhost Resistance of *Thinopyrum ponticum* to *Puccinia graminis* f. sp. *tritici* and the Effects of the *Sr24*, *Sr25*, and *Sr26* Genes Introgressed to Wheat. *Int. J. Plant Biol.* 14, 435–457. doi: 10.3390/ijpb14020034

Plotnikova, L., Sagendykova, A., and Pozherukova, V. (2023b). The Use of Genetic Material of Tall Wheatgrass to Protect Common Wheat from Septoria Blotch in Western Siberia. *Agriculture* 13, 203. doi: 10.3390/agriculture13010203

Qonaah, I. A., Simon, A. L., Warner, D., Bruce, T. J. A., and Ray, R. V. (2026). An aphid‐resistant wheat variety reduces the transmission of barley yellow dwarf virus (BYDV) by *Rhopalosiphum padi* (L.). *Pest Manag. Sci.* 82, 4849–4859. doi: 10.1002/ps.70599

Salina, E. A., Adonina, I. G., Badaeva, E. D., Kroupin, P. Yu., Stasyuk, A. I., Leonova, I. N., et al. (2015). A *Thinopyrum intermedium* chromosome in bread wheat cultivars as a source of genes conferring resistance to fungal diseases. *Euphytica* 204, 91–101. doi: 10.1007/s10681-014-1344-5

Sears, E. R. (1973). *Agropyron*-wheat transfers induced by homoeologous pairing. In *Proceedings of the Fourth International Wheat Genetics Symposium. Alien Genetic Material*, 191–199.

Sharp, G. L., Martin, J. M., Lanning, S. P., Blake, N. K., Brey, C. W., Sivamani, E., et al. (2002). Field Evaluation of Transgenic and Classical Sources of *Wheat streak mosaic virus* Resistance. *Crop Sci.* 42, 105–110. doi: 10.2135/cropsci2002.1050

Shchuklina, O., Alenicheva, A., Samokhina, V., Voronchikhina, I., Shchelkanov, D., Demchuk, N., et al. (2026). Morphological and Baking Properties of the Blue-Grained ×*Trititrigia cziczinii* Tzvelev Line ‘Istra 116’: A New Donor for Wheat Anthocyanin Biofortification. *Crops* 6, 19. doi: 10.3390/crops6010019

Silva, P., Evers, B., Kieffaber, A., Wang, X., Brown, R., Gao, L., et al. (2022). Applied phenomics and genomics for improving barley yellow dwarf resistance in winter wheat. *G3 Genes Genomes Genet.* 12, jkac064. doi: 10.1093/g3journal/jkac064

Simonov, A. V., Gordeeva, E. I., Genaev, M. A., Li, W., Bulatov, I. O., and Pshenichnikova, T. A. (2024). A new leaf pubescence gene, *Hl1th*, introgressed into bread wheat from *Thinopyrum ponticum* and its phenotypic manifestation under homoeologous chromosomal substitutions. *Vavilov J. Genet. Breed.* 28, 602–609. doi: 10.18699/vjgb-24-67

Sivasamy, M., Jayaprakash, P., Vikas, V. K., Bharadwaj, S. C., C., M., R, N., et al. (2022). Effectiveness of *Thinopyrum ponticum*-derived wheat leaf rust resistance gene, *Lr24* in India - a revisit. *J. Cereal Res.* 14. doi: 10.25174/2582-2675/2022/129000

Smith, E. L., Schlehuber, A. M., Young, H. C., and Edwards, L. H. (1968). Registration of Agent Wheat¹ (Reg. No. 471). *Crop Sci.* 8, 511–512. doi: 10.2135/cropsci1968.0011183X000800040039x

Song, R., Cheng, Y., Wen, M., Song, X., Wang, T., Xia, M., et al. (2023). Transferring a new Fusarium head blight resistance locus *FhbRc1* from *Roegneria ciliaris* into wheat by developing alien translocation lines. *Theor. Appl. Genet.* 136, 36. doi: 10.1007/s00122-023-04278-1

Song, R., Zhang, D., Yang, J., Cheng, Y., Song, X., Zhao, W., et al. (2024). Identification and transferring of a new Fusarium head blight resistance gene *FhbRc2* from *Roegneria ciliaris* 3ScL chromosome arm into common wheat. *Crop J.* 12, 1718–1726. doi: 10.1016/j.cj.2024.07.010

Tian, H., Mu, Y., Yang, S., Zhang, J., Yang, X., Zhang, Q., et al. (2024). ATAC sequencing and transcriptomics reveal the impact of chromatin accessibility on gene expression in *Tritipyrum* under salt-stress conditions. *Environ. Exp. Bot.* 228, 106014. doi: 10.1016/j.envexpbot.2024.106014

Tounsi, S., Giorgi, D., Kuzmanović, L., Jrad, O., Farina, A., Capoccioni, A., et al. (2024). Coping with salinity stress: segmental group 7 chromosome introgressions from halophytic *Thinopyrum* species greatly enhance tolerance of recipient durum wheat. *Front. Plant Sci.* 15, 1378186. doi: 10.3389/fpls.2024.1378186

Türkösi, E., Kruppa, K., Darkó, É., Varga, B., György, M., Gulyás, Z., et al. (2025). Replacement of chromosome 3D with *Thinopyrum* chromosome 3St led to increased drought tolerance during the flowering stage in wheat. *Plant Cell Rep.* 44, 242. doi: 10.1007/s00299-025-03632-5

Wang, H., Sun, S., Ge, W., Zhao, L., Hou, B., Wang, K., et al. (2020a). Horizontal gene transfer of *Fhb7* from fungus underlies Fusarium head blight resistance in wheat. *Science* 368, eaba5435. doi: 10.1126/science.aba5435

Wang, X., Gong, W., Zhang, R., Wu, J., Yan, H., He, H., et al. (2025). Creation of a Stripe Rust- and Leaf Rust-Resistant Wheat–*Elymus trachycaulus* Translocation Line with Reduced 1Ht^S^ Alien Chromatin. *Phytopathology* 115, 1477–1485. doi: 10.1094/PHYTO-08-24-0264-R

Wang, Y., Cao, Q., Zhang, J., Wang, S., Chen, C., Wang, C., et al. (2020b). Cytogenetic Analysis and Molecular Marker Development for a New Wheat–*Thinopyrum ponticum* 1Js (1D) Disomic Substitution Line With Resistance to Stripe Rust and Powdery Mildew. *Front. Plant Sci.* 11, 1282. doi: 10.3389/fpls.2020.01282

Wei, Y., Zhang, T., Jin, Y., Li, W., Kong, L., Liu, X., et al. (2024). Introgression of an adult-plant powdery mildew resistance gene *Pm4VL* from *Dasypyrum villosum* chromosome 4V into bread wheat. *Front. Plant Sci.* 15, 1401525. doi: 10.3389/fpls.2024.1401525

Wells, D. G., Kota, R., Sandhu, H., Gardner, W., and Finney, K. (1982). Registration of one disomic substitution line and five translocation lines of winter wheat germplasm resistant to wheat streak mosaic virus (Reg. No. GP 199 to GP 204). *Crop Sci.* 22, 1277–1278. doi: 10.2135/cropsci1982.0011183X002200060083x

Wu, C., Zhao, X., Li, M., Qu, Y., Yan, X., Ding, J., et al. (2025). Development and molecular cytogenetic characterization of black-grain wheat derived from wheat-*Thinopyrum intermedium* hybridization. *Theor. Appl. Genet.* 138, 184. doi: 10.1007/s00122-025-04968-y

Xing, L., Hu, P., Liu, J., Witek, K., Zhou, S., Xu, J., et al. (2018). *Pm21* from *Haynaldia villosa* Encodes a CC-NBS-LRR Protein Conferring Powdery Mildew Resistance in Wheat. *Mol. Plant* 11, 874–878. doi: 10.1016/j.molp.2018.02.013

Xu, S., Lyu, Z., Zhang, N., Li, M., Wei, X., Gao, Y., et al. (2023). Genetic mapping of the wheat leaf rust resistance gene *Lr19* and development of translocation lines to break its linkage with yellow pigment. *Theor. Appl. Genet.* 136, 200. doi: 10.1007/s00122-023-04425-8

Yang, G., Zhang, N., Boshoff, W. H. P., Li, H., Li, B., Li, Z., et al. (2023). Identification and introgression of a novel leaf rust resistance gene from *Thinopyrum intermedium* chromosome 7Js into wheat. *Theor. Appl. Genet.* 136, 231. doi: 10.1007/s00122-023-04474-z

Yang, R., Yang, Z., Peng, Z., He, F., Shi, L., Dong, Y., et al. (2021). Integrated transcriptomic and proteomic analysis of *Tritipyrum* provides insights into the molecular basis of salt tolerance. *PeerJ* 9, e12683. doi: 10.7717/peerj.12683

Ye, X., Zhang, S., Li, S., Wang, J., Chen, H., Wang, K., et al. (2019). Improvement of three commercial spring wheat varieties for powdery mildew resistance by marker-assisted selection. *Crop Prot.* 125, 104889. doi: 10.1016/j.cropro.2019.104889

Yin, X., Shang, X., Pang, B., Song, J., Cao, S., Li, J., et al. (2006). Molecular Mapping of Two Novel Stripe Rust Resistant Genes *YrTp1* and *YrTp2* in A-3 Derived from *Triticum aestivum* × *Thinopyrum ponticum*. *Agric. Sci. China* 5, 483–490. doi: 10.1016/S1671-2927(06)60081-3

Yu, G., Matny, O., Gourdoupis, S., Rayapuram, N., Aljedaani, F. R., Wang, Y. L., et al. (2023). The wheat stem rust resistance gene *Sr43* encodes an unusual protein kinase. *Nat. Genet.* 55, 921–926. doi: 10.1038/s41588-023-01402-1

Zeng, J., Zhou, C., He, Z., Wang, Y., Xu, L., Chen, G., et al. (2023). Disomic Substitution of 3D Chromosome with Its Homoeologue 3E in Tetraploid *Thinopyrum elongatum* Enhances Wheat Seedlings Tolerance to Salt Stress. *Int. J. Mol. Sci.* 24, 1609. doi: 10.3390/ijms24021609

Zhang, J., Jie, Y., Yan, L., Wang, M., Dong, Y., Pang, Y., et al. (2024). Development and identification of a novel wheat-*Thinopyrum ponticum* disomic substitution line DS5Ag(5D) with new genes conferring resistance to powdery mildew and leaf rust. *BMC Plant Biol.* 24, 718. doi: 10.1186/s12870-024-05433-6

Zhang, J., Tang, S., Lang, T., Wang, Y., Long, H., Deng, G., et al. (2022a). Molecular Cytogenetic Identification of the Wheat–*Dasypyrum villosum* T3DL·3V#3S Translocation Line with Resistance against Stripe Rust. *Plants* 11, 1329. doi: 10.3390/plants11101329

Zhang, R., Fan, Y., Kong, L., Wang, Z., Wu, J., Xing, L., et al. (2018). *Pm62*, an adult-plant powdery mildew resistance gene introgressed from *Dasypyrum villosum* chromosome arm 2VL into wheat. *Theor. Appl. Genet.* 131, 2613–2620. doi: 10.1007/s00122-018-3176-5

Zhang, R., Feng, Y., Li, H., Yuan, H., Dai, J., Cao, A., et al. (2016). Cereal cyst nematode resistance gene *CreV* effective against *Heterodera filipjevi* transferred from chromosome 6VL of *Dasypyrum villosum* to bread wheat. *Mol. Breed.* 36, 122. doi: 10.1007/s11032-016-0549-9

Zhang, R., Lu, C., Meng, X., Fan, Y., Du, J., Liu, R., et al. (2022b). Fine mapping of powdery mildew and stripe rust resistance genes *Pm5V*/*Yr5V* transferred from *Dasypyrum villosum* into wheat without yield penalty. *Theor. Appl. Genet.* 135, 3629–3642. doi: 10.1007/s00122-022-04206-9

Zhang, R., Xiong, C., Mu, H., Yao, R., Meng, X., Kong, L., et al. (2021). *Pm67*, a new powdery mildew resistance gene transferred from *Dasypyrum villosum* chromosome 1V to common wheat (*Triticum aestivum* L.). *Crop J.* 9, 882–888. doi: 10.1016/j.cj.2020.09.012

Zhang, W., Danilova, T., Zhang, M., Ren, S., Zhu, X., Zhang, Q., et al. (2022c). Cytogenetic and genomic characterization of a novel tall wheatgrass-derived *Fhb7* allele integrated into wheat B genome. *Theor. Appl. Genet.* 135, 4409–4419. doi: 10.1007/s00122-022-04228-3

Zhao, J., Liu, Y., Cheng, X., Pang, Y., Li, J., Su, Z., et al. (2019). Development and identification of a dwarf wheat-*Leymus mollis* double substitution line with resistance to yellow rust and Fusarium head blight. *Crop J.* 7, 516–526. doi: 10.1016/j.cj.2018.11.012

Zhao, L., Bernardo, A., Kong, F., Zhao, W., Dong, Y., Lee, H., et al. (2024). A Glutathione S-Transferase from *Thinopyrum ponticum* Confers *Fhb7* Resistance to Fusarium Head Blight in Wheat. *Phytopathology* 114, 1458–1461. doi: 10.1094/PHYTO-03-24-0106-SC

Zhao, W., Zhao, L., Fellers, J., Bowden, R., Xu, S., and Bai, G. (2025). Development and validation of diagnostic markers for wheat stem rust resistance gene *Sr43*. *Crop Sci.* 65, e70132. doi: 10.1002/csc2.70132

Zheng, X., Tang, C., Han, R., Zhao, J., Qiao, L., Zhang, S., et al. (2020). Identification, Characterization, and Evaluation of Novel Stripe Rust-Resistant Wheat–*Thinopyrum intermedium* Chromosome Translocation Lines. *Plant Dis.* 104, 875–881. doi: 10.1094/PDIS-01-19-0001-RE

Zhong, G.-Y., and Dvořák, J. (1995). Evidence for common genetic mechanisms controlling the tolerance of sudden salt stress in the tribe Triticeae. *Plant Breed.* 114, 297–302. doi: 10.1111/j.1439-0523.1995.tb01237.x
